# α-PD-1 therapy elevates Treg/Th balance and increases tumor cell pSmad3 that are both targeted by α-TGFβ antibody to promote durable rejection and immunity in squamous cell carcinomas

**DOI:** 10.1186/s40425-018-0493-9

**Published:** 2019-03-04

**Authors:** E. Dodagatta-Marri, D. S. Meyer, M. Q. Reeves, R. Paniagua, M. D. To, M. Binnewies, M. L. Broz, H. Mori, D. Wu, M. Adoumie, R. Del Rosario, O. Li, T. Buchmann, B. Liang, J. Malato, F. Arce Vargus, D. Sheppard, B. C. Hann, A. Mirza, S. A. Quezada, M. D. Rosenblum, M. F. Krummel, A. Balmain, R. J. Akhurst

**Affiliations:** 1 Helen Diller Family Comprehensive Cancer Center, UCSF, San Francisco, CA USA; 2 Department of Dermatology, UCSF, San Francisco, CA USA; 3 Department of Pathology, UCSF, San Francisco, CA USA; 4 Center for Comparative Medicine UC Davis, Davis, CA USA; 5 Department of Medicine, UCSF, San Francisco, CA USA; 6 Xoma Corporation, Berkeley, CA USA; 7 Cancer Immunology Unit, Immune Regulation and Tumour Immunotherapy Lab, University College London, London, UK; 8 UCSF Parker Institute for Cancer Immunotherapy, San Francisco, CA USA; 9 Department of Biochemistry and Biophysics, UCSF, San Francisco, CA USA; 10 Department of Anatomy, UCSF, San Francisco, CA USA

**Keywords:** Checkpoint blockade, Squamous cell carcinoma, Tumor mutation load, α-TGFβ /α-PD-1 combinatorial immunotherapy, Tregs, pSmad signaling, Epithelial mesenchymal transition (EMT)

## Abstract

**Background:**

Checkpoint blockade immunotherapy has improved metastatic cancer patient survival, but response rates remain low. There is an unmet need to identify mechanisms and tools to circumvent resistance. In human patients, responses to checkpoint blockade therapy correlate with tumor mutation load, and intrinsic resistance associates with pre-treatment signatures of epithelial mesenchymal transition (EMT), immunosuppression, macrophage chemotaxis and TGFβ signaling.

**Methods:**

To facilitate studies on mechanisms of squamous cell carcinoma (SCC) evasion of checkpoint blockade immunotherapy, we sought to develop a novel panel of murine syngeneic SCC lines reflecting the heterogeneity of human cancer and its responses to immunotherapy. We characterized six Kras-driven cutaneous SCC lines with a range of mutation loads. Following implantation into syngeneic FVB mice, we examined multiple tumor responses to α-PD-1, α-TGFβ or combinatorial therapy, including tumor growth rate and regression, tumor immune cell composition, acquired tumor immunity, and the role of cytotoxic T cells and Tregs in immunotherapy responses.

**Results:**

We show that α-PD-1 therapy is ineffective in establishing complete regression (CR) of tumors in all six SCC lines, but causes partial tumor growth inhibition of two lines with the highest mutations loads, CCK168 and CCK169. α-TGFβ monotherapy results in 20% CR and 10% CR of established CCK168 and CCK169 tumors respectively, together with acquisition of long-term anti-tumor immunity. α-PD-1 synergizes with α-TGFβ, increasing CR rates to 60% (CCK168) and 20% (CCK169). α-PD-1 therapy enhances CD4 + Treg/CD4 + Th ratios and increases tumor cell pSmad3 expression in CCK168 SCCs, whereas α-TGFβ antibody administration attenuates these effects. We show that α-TGFβ acts in part through suppressing immunosuppressive Tregs induced by α-PD-1, that limit the anti-tumor activity of α-PD-1 monotherapy. Additionally, in vitro and in vivo, α-TGFβ acts directly on the tumor cell to attenuate EMT, to activate a program of gene expression that stimulates immuno-surveillance, including up regulation of genes encoding the tumor cell antigen presentation machinery.

**Conclusions:**

We show that α-PD-1 not only initiates a tumor rejection program, but can induce a competing TGFβ-driven immuno-suppressive program. We identify new opportunities for α-PD-1/α-TGFβ combinatorial treatment of SCCs especially those with a high mutation load, high CD4+ T cell content and pSmad3 signaling. Our data form the basis for clinical trial of α-TGFβ/α-PD-1 combination therapy (NCT02947165).

**Electronic supplementary material:**

The online version of this article (10.1186/s40425-018-0493-9) contains supplementary material, which is available to authorized users.

## Background

Antibody-based therapies targeting T cell checkpoint receptors, CTLA-4 and PD-1, have ushered in a revitalized era of cancer immunotherapy (IMT). α-PD-1 mediated immune checkpoint blockade is now first line therapy for melanoma and PD-L1+ lung cancer, and second line therapy for squamous cell carcinoma of the head and neck (SCC-HN), and other tumor types [1, 2]. However, response rates vary across tumor types, and even within the most responsive cancer, namely melanoma, durable responses occur in only a minority of patients and 25% of responding patients eventually relapse [1, 2]. Importantly, α-PD-1 therapy can paradoxically accelerate tumor growth in certain patients by mechanisms that are presently unclear [3–5]. Many efforts are therefore underway to increase α-PD-1 response rates, identify mechanisms of intrinsic and acquired α-PD-1 drug resistance, predict potential responders or super-progressors prior to treatment, and investigate new combinatorial drug regimens.

A major correlate of clinical response to immune checkpoint blockade is the quantitative load of somatic non-synonymous single nucleotide mutations (NS-SNMs) [6]. We therefore utilized a mouse model of chemically-induced cutaneous squamous cell carcinomas (cSCC) which have a range of somatic mutations that mimic the NS-SNM mutation burden of environmentally-induced human cancers [7]. In this cSCC model, endogenous *Hras* or *Kras* oncogenic drivers are chemically-activated by local 7,12-dimethylbenz (*a*) anthracene (DMBA) treatment that induces transversion mutations resulting primarily in *KrasG13R* or *HrasQ61L* somatic mutations [7]. Subsequent tumor outgrowth depends on repeated exposure to the inflammation-inducing phorbol ester, 12–*0*-tetradecanoyl-phorbol-13 acetate, thus mimicking the important role of chronic inflammation in development of many human cancers. This two-step chemical carcinogenesis model has been thoroughly characterized with respect to dominant driving genetic events, evolution of the mutational landscape as carcinomas progress from initiation through benign and malignant stages, and in vivo epithelial-to-mesenchymal transition (EMT) and metastasis [7–9]. The biphasic activities of TGFβ during tumor progression and synergy between Ras and TGFβ have also been intensively studied using this model [8, 10–12].

In humans, intrinsic resistance of melanoma to α-PD-1 therapy has been associated with a transcriptomic signature enriched for markers of EMT, immune suppression and macrophage chemotaxis [13], all of which can be driven by TGFβ signaling [11, 12, 14, 15]. Moreover, recent genomic and transcriptomic analysis of a large panel of human urothelial carcinomas showed a positive association between tumor mutation load (TML) and clinical response to the checkpoint inhibitor, atezolizumab, an antibody that blocks a ligand for the PD-1 receptor, PDL-1, [16]. This study also demonstrated that in the subset of bladder carcinomas that are “immune-excluded”, the strongest pre-treatment transcriptomic signature that associates with failure to respond to subsequent atezolizumab therapy is enrichment for a fibroblastic signature with high expression of TGFβ signaling genes particularly *TGFB1* and *TGFBR2* [16]. This, and another study of colon carcinomas [17], concluded that TGFβ signaling within cancer-associated fibroblasts (CAFs) forms a barrier to intra-tumoral penetration of immune cells that can be alleviated by blockade of TGFβ signaling, resulting in synergy between α-PDL-1 and α-TGFβ therapy. Additional studies have reported additive, synergistic or redundant anti-tumor interactions between TGFβ signaling and PD-1/PD-L1 blockade in different model systems in vitro and in vivo [18–22].

Herein, we generated a number of cutaneous SCC tumor lines derived from chemically-induced primary carcinomas and from the low mutation load genetically-engineered mouse model (GEMM), *Lgr5-Cre-ERT2* x *Kras*^*G12D*^*-LSL* [23]. In agreement with observations on human cancers [6, 16, 24], we found that the SCC lines with highest TMLs are the most responsive to α-PD-1, but even in these high TML SCCs, α-PD-1 therapy rarely achieves complete regression (CR). We find that in high TML SCCs, α-PD-1 therapy further elevates tumor cell pSmad3 signaling and increases the fraction of CD4+ T cells that are immunosuppressive Tregs (Foxp3 + CD25+), thus restraining the anti-tumor immune response to this checkpoint inhibitor, but a combination of α-TGFβ with α-PD-1 synergistically enhances anti-tumor responses. We show that drug synergy is driven by induction, not only of T effector cell activation by α-PD-1, but of a competing TGFβ-driven immunosuppressive program that acts to induce tumor cell EMT and polarization of CD4+ T cells to blunt the response to α-PD-1 therapy.

## Methods

Detailed methods and statistical tests can be found in Additional file 1: Supplementary Methods.

## Results

### α-PD-1 monotherapy elevates immunosuppressive Tregs in chemically induced squamous carcinomas

**Fig. 1:**
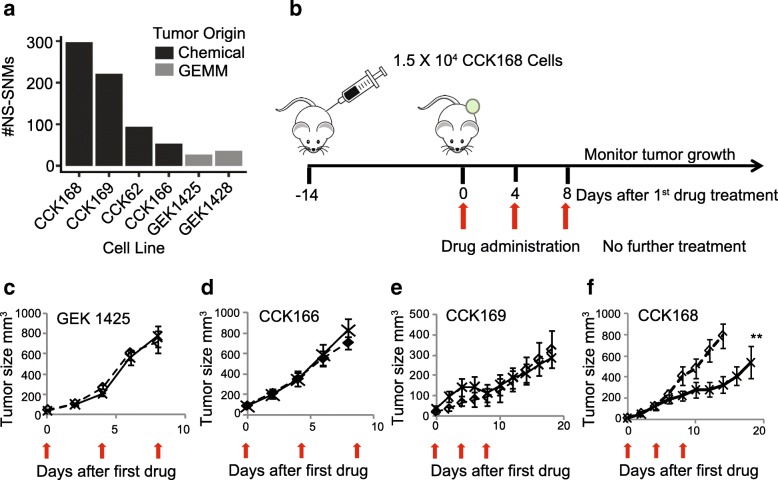
α-PD-1 effects on tumor-growth of chemically-induced and GEMM-derived SCCs. **a** Total NS-SNV loads of *Kras*-driven SCCs, determined by WES analysis. The panel includes four chemically-induced (CCK) and two GEMM-derived (GEK) SCCs, **b** Scheme for syngeneic tumor generation and drug therapy: After implantation of 1.5 × 10^4^ tumor cells by unilateral dorsolateral subcutaneous injection, tumors grew for 14 days until they reached ≥5 mm diameter. Mice were then treated with control IgGs or α-PD-1 drugs injected intraperitoneally (ip) into the contralateral side, with three drug administrations, each four days apart (considered days 0, 4 and 8). **c**-**f**) Tumor growth of the indicated tumor lines with or without α-PD-1 therapy was measured at least every other day from the time of first drug administration. Red arrows indicate timing of drug administration (see Additional file 3: Figure S1 for GEK1428, and CCK62 growth curves), **c**-**e**) mean tumor growth of 7–10 mice per arm. ** = p < 0.01 (Fisher’s Exact test)

We first generated a panel of highly aggressive *Kras-*driven cSCC, four derived from DMBA-initiated *Kras*^*G13R*^ mutant tumors and two derived from tumors of the GEMM, *Lgr5-Cre-ERT2* x *Kras*^*G12D*^*-LSL* [23], all on the FVB/NJ strain background. Whole exome sequencing (WES) revealed that the chemically induced tumor lines represent a range of mutation loads, from ~ 50 to 300 NS-SNMs per exome (Fig. 1a, Additional file 2: Table S1), similar to that of human cancer [6, 25], but the two GEMM-derived tumor lines that are driven by a transgenic *Kras*^*G12D*^ oncogene had less than 40 NS-SNMs per exome (Fig. 1a, Additional file 2: Table S1).

In most other syngeneic mouse models, α-PD-1 as monotherapy has no effect on primary tumor outgrowth [26, 27]. Consistent with this, when administered intra-peritoneally (*ip*) to mice with established tumors (Fig. 1b), most of the SCC lines show little or no tumor growth response to α-PD-1, including the two GEMMs and three of the chemically-induced cSCCs, CCK62, CCK166 and CCK169 (Fig. 1 c-e, and Additional file 3: Figure S1). In contrast, CCK168, which has the highest TML, shows a significant delay in tumor outgrowth in response to α-PD-1 monotherapy (Figs. 1f), This finding is compatible with data from human clinical trials that show a positive relationship between TML and response to checkpoint blockade inhibitors [6, 16, 24].

**Fig. 2:**
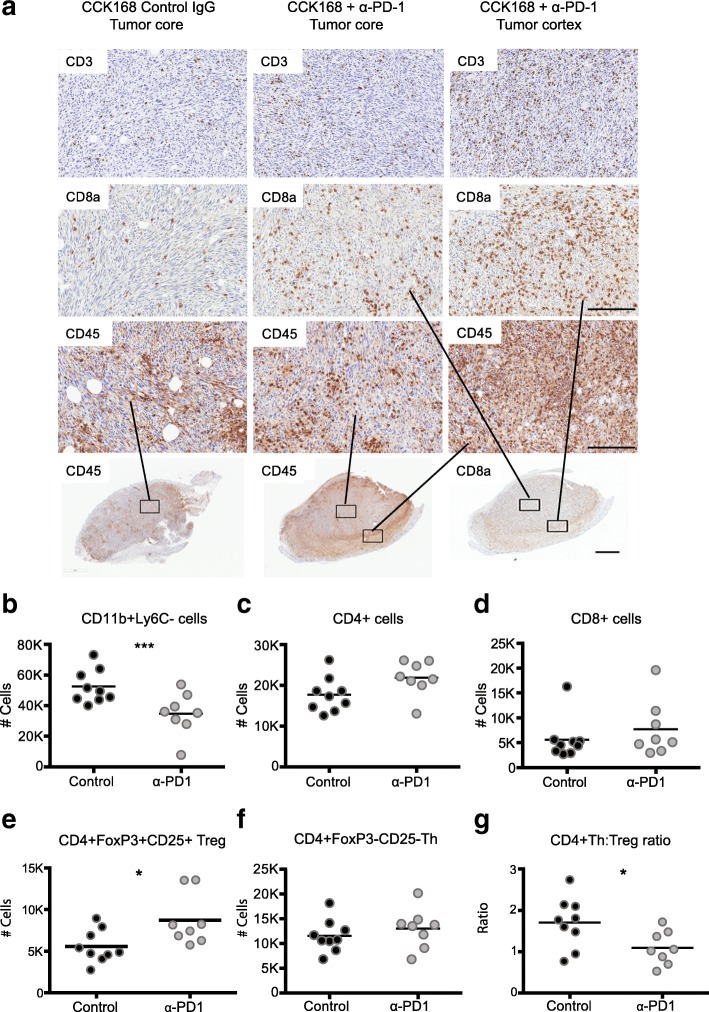
Alteration in tumor infiltrating leukocyte profiles in response to α-PD-1 therapy. Tumors, generated according to the scheme in Fig. 1b, were harvested 7 days after the first drug treatment and analyzed by (**a**) immunohistochemistry or (**b**-**g**) 11-color flow cytometry. **a** Immunohistochemical staining shows CD3+ total T cells, CD8a + cytotoxic T cells and CD45+ immune cells in the non-responsive tumor line, CCK62, and in the responsive tumor line, CCK168. **b**-**g** Flow cytometric analysis of CCK168 immune cell subsets in response to α-PD-1 therapy: **b** CD11b + Gr1-Ly6C-Ly6Clo myeloid cells, **c** total CD4+ T cells, **d** cytotoxic CD8+ T cells, **e** CD4 + CD25 + Foxp3+ Treg cells, **f** CD4 + Foxp3-CD25- Th cells, **g** ratio of CD4+ Th/Treg cells. Cell numbers shown are relative to 100, 000 CD45+ immune cells. Representative data of ≥ three biological replicates. Scale bars in (a) represents 50 μM in upper panels and 200 μM in lower panel. * = p < 0.05; ** = p < 0.01; *** = p < 0.001: Mann Whitney U test

We next investigated changes in intra-tumoral immune cell subsets in CCK168 tumors, seven to eight days after initial α-PD-1 treatment, at a time when responsive tumors begin to diminish in size (see Fig. 1f). Immunohistochemical (IHC) analysis suggested only a slight increase in CD45+, CD3+, and CD8+ T cell infiltration into CCK168 tumors after α-PD-1 monotherapy, and the majority of infiltrating immune cells did not fully penetrate the tumor core but were located within the outer cortex of the tumor parenchyma (Fig. 2a). Using multicolor flow cytometry, we found that around 50% of CD45+ tumor infiltrating leukocytes were Cd11b+ myeloid cells (Fig. 2b), and CD4+ T cells were the most common T lymphocyte cell type in IgG-treated CCK168 control carcinomas. Total CD4+ T cells constitute 20% of tumor infiltrating CD45+ leukocytes (Fig. 2c), whereas CD8+ cytotoxic T cells contribute only 5% of the leukocyte population (Fig. 2d). α-PD-1 therapy caused a significant decrease in MHCII+ myeloid cell numbers (Fig. 2b), driven by a reduction in the major myeloid population of Ly6C^lo^ tumor associated macrophages (TAMs), that are considered immuno-suppressive [28].

As determined by flow cytometry, α-PD-1 monotherapy did not significantly affect tumor infiltration of total CD4+ (Fig. 2c) or CD8+ T cells (Fig. 2d) but, unexpectedly, it consistently and significantly skewed the balance between CD4 + CD25 + Foxp3+ Tregs and CD4 + Foxp3- Th cells (Fig. 2 e-g). Bearing in mind the prominence of CD4+ T cells within the lymphocyte population of CCK168 tumors, we considered that the enhanced CD4 + Treg to CD4 + Teff (effector) ratio (Fig. 2g), and the presumed change in cytokine profile from inflammatory to immunosuppressive T cells, might contribute in part to reduced penetration of cytotoxic CD8+ T cells into the tumor core.

### α-TGFβ monotherapy initiates durable CR of SCCs and synergizes with α-PD-1 therapy

**Fig. 3:**
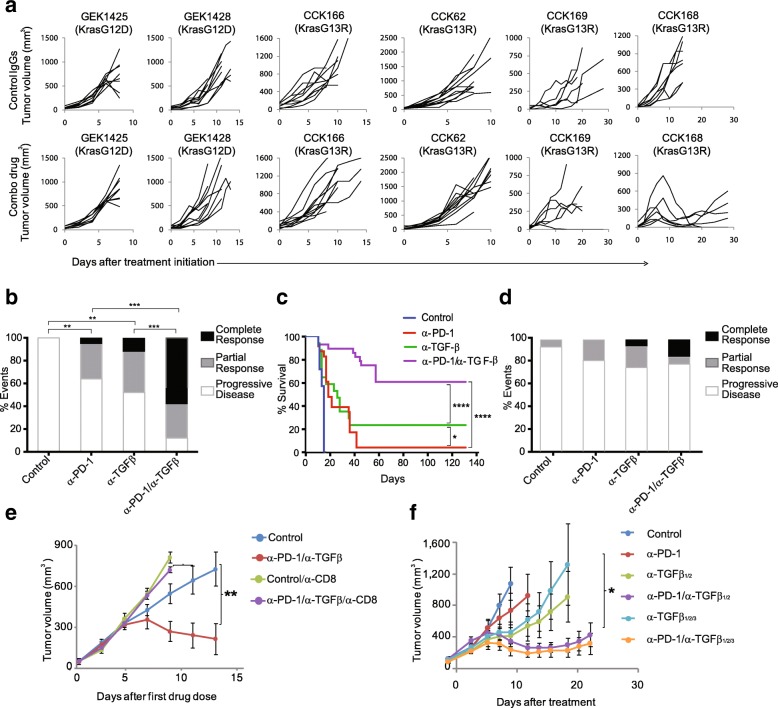
α-TGFβ and α-PD-1 synergize in eliciting tumor rejection in a CD8a + cell-dependent manner. **a** Growth curves of each of the six SCC lines, as indicated, after treatment with control IgG or α-TGFβ/α-PD-1 combination therapy on days 0, 4 and 8 (*n* = 8–10 mice per arm). Tumors were measured every other day after tumor implantation and therapy (see Fig. 1b). Summary of tumor responses for CCK168 (**b**) and CCK169 (**d**), and overall survival for CCK168 (**c**) following treatment on days 0, 4 and 8 with control IgG, α-PD-1, α-TGFβ or combination therapy. In (**b**) and (**d**), individual tumors were classified as complete responders (CR), partial responders (PR), or progressive disease (PD), according to their growth characteristics (CR, tumor eradicated with no regrowth; PR, tumor shrinkage ≥30%; PD, no effect of drugs compared to control IgG). For each drug arm, the percentages of total tumors within each response group are shown. *n* = 10 mice per arm per experiment, *n* = 3 independent experiments. **c** Kaplan Meier survival plot for mice bearing CCK168 tumors, after treatment on days 0, 4 and 8 with the indicated drugs. 2000 mm3 tumor size was used as the cut off for survival. **e** Average CCK168 tumor growth curves following treatment with α-PD-1/α-TGFβ combination therapy versus isotype control IgGs in mice with or without CD8+ T cell depletion. Animals were treated as in Fig. 1b, except that 24 h prior to the first therapeutic drug dose, mice received *ip* injection of CD8a + cell-depleting antibody or isotype matched control IgG. f) CCK168 tumor-bearing mice were administered one of six IgG combinations, either human α-pan-TGFβ IgG2 (XPA.42.068), human α-TGFβ1/β2 IgG2 (XPA.42.089) or human α-keyhole limpet hemocyanin as control IgG2, together with rat α-PD-1 IgG2 or rat IgG2 control, on days 0, 4 and 8. Average tumor volume (mm^3^) +/− SD. * = p < 0.05; ** = p < 0.01; *** = p < 0.001: Fisher’s exact test (b), Gehan-Breslow-Wilcoxon test (c), Student’s test (e, f)

iTreg differentiation is driven by the master transcription factor, Foxp3, which is transcriptionally activated by TGFβ effectors, pSmad3 and Smad4 [29], we thus postulated that suppression of Treg activation through blockade of TGFβ ligands might enhance α-PD-1 tumor responses. Moreover, several recent studies gave credence to the possibility of synergy between TGFβ inhibition and blockade of the PD-1/PDL-1 axis [16–19]. We therefore utilized a pan-TGF-β neutralizing antibody, XPA-42-068 [30] that blocks all three activated TGFβ ligands, to test for drug synergy in the various SCC lines. We tested each of the six *Kras*-driven tumor lines for responses to the combination of α-PD-1 and α-TGFβ antibodies (Fig. 3a). Both CCK168 and CCK169, each with NS-SNM loads greater than 200 (Fig. 1a), showed highly heterogeneous tumor responses following administration of both drugs, with cases of progressive disease as well as partial or complete tumor responses (Fig. 3 a-d). The other SCC lines, all of which have NS-SNM loads less than 100 (Fig. 1a), lacked responses to the drug combination (Fig. 3a). Surprisingly, the α-TGFβ antibody given as monotherapy showed significantly better anti-tumor efficacy than α-PD-1 monotherapy for CCK168 tumors (Fig. 3 b, c), with more than 20% of established CCK168 tumors showing durable CR after α-TGFβ monotherapy (Fig. 3c). A similar trend was observed in the CCK169 model (Fig. 3d). Importantly, α-TGFβ synergizes with α-PD-1 resulting in 60% overall long-term survival in the CCK168 model and 20% CR for CCK169 (Figs. 3 b-d, Additional file 3: Figure S2).

To test the requirement for adaptive immune responses in eliciting SCC tumor regression, we depleted cytotoxic CD8+ T cells using an α-CD8a antibody that induces antibody-dependent cellular cytotoxicity (ADCC). We found that the effect of combinatorial therapy on CCK168 tumors was completely abolished (Fig. 3e). Moreover, consistent with ongoing immune surveillance, CCK168 tumor growth accelerated after CD8+ T cell depletion, regardless of the presence of immunotherapy drugs (Fig. 3e). It is possible that some of the effects of the α-CD8a antibody may be mediated by depletion of CD8+ DCs that play a unique role in cross-presentation of tumor antigens on MHC class I [31]. Regardless, the data support the conclusion that α-TGFβ-induced tumor regression is predominantly mediated through adaptive immunity.

Finally, we compared the efficacy of the α-pan-TGFβ antibody with that of a clinical lead, currently in phase Ib trial in combination with α-PD-1. This antibody, XPA-42-089, that blocks TGFβ1 and TGFβ2, but cannot bind and block TGFβ3, was as efficacious as XPA-42-068 as monotherapy or when combined with α-PD-1 (Fig. 3f). TGFβ3 therefore does not appear to play a major immunosuppressive role in the CCK168 SCC model.

### α-TGFβ drives long-term tumor immunity against KrasG13R driven SCCs

Since CD8+ cytotoxic T cells drive tumor rejection, we investigated long-term immunity to tumor re-challenge. Indeed, parental CCK168 cells failed to grow when implanted into “CCK168-cured” mice (*n* > 24) despite robust growth when implanted into tumor-naïve mice (*n* = 3–5 tumor-naïve mice per test Fig. S3), suggesting activation of immune memory to CCK168 by TGFβ blockade. This immune memory of CCK168-cured mice persists even 18 months beyond the last drug dose. Intriguingly, CCK169 or CCK166 cells, when implanted into “CCK168-cured” mice, also failed to grow despite robust growth in tumor-naïve mice (Fig. S3). This was despite the latter tumor line showing complete resistance to de novo α-TGFβ/α-PD-1 combination therapy (Fig. 3a). WES analysis revealed that the chemically-induced *KrasG13R* mutation was the only potential neoantigen shared between CCK166, CCK168 and CCK169 (Additional file 4: Table S2)*,* raising the possibility that, in this model, *KrasG13R* is a dominant neoantigen driving immune rejection. Notably, in human cancer, mutation of *KRAS-G12D* has been shown to generate a neoantigen that elicits CD8+ cytotoxic T cell responses [32].

Supporting the concept that *KrasG13R* encodes a dominant neoepitope in the CCK168 model, a chemically-induced *HrasQ61L-*driven SCC, CCH85 (214 NS-SNMs), grew equally well when implanted into either tumor-naive or “CCK168-cured” mice (Fig. S3). Moreover, three of six “CCK168-cured” mice supported robust tumor growth of implanted *KrasG12D*-driven GEK1425 or GEK1428 tumor cells, further supporting a requirement for the *KrasG13R* neoepitope in driving tumor rejection. However, three other “CCK168-cured” mice rejected outgrowth of GEK1425 or GEK1428 tumor cells indicating that additional minor tumor epitopes, shared between these *KrasG12D*-driven tumor lines and *KrasG13R*-driven CCK168, can also elicit tumor rejection. Such common epitopes in these *Kras*-driven tumors may result from quantitatively abnormal protein expression or splicing [18, 33] that would be undetectable by WES analysis. Further studies would be required to demonstrate that *KrasG13R* is a dominant neoantigen in this mouse model.

### Synergy between α-TGFβ and α-PD-1 is mediated in part through effects on Tregs

**Fig. 4:**
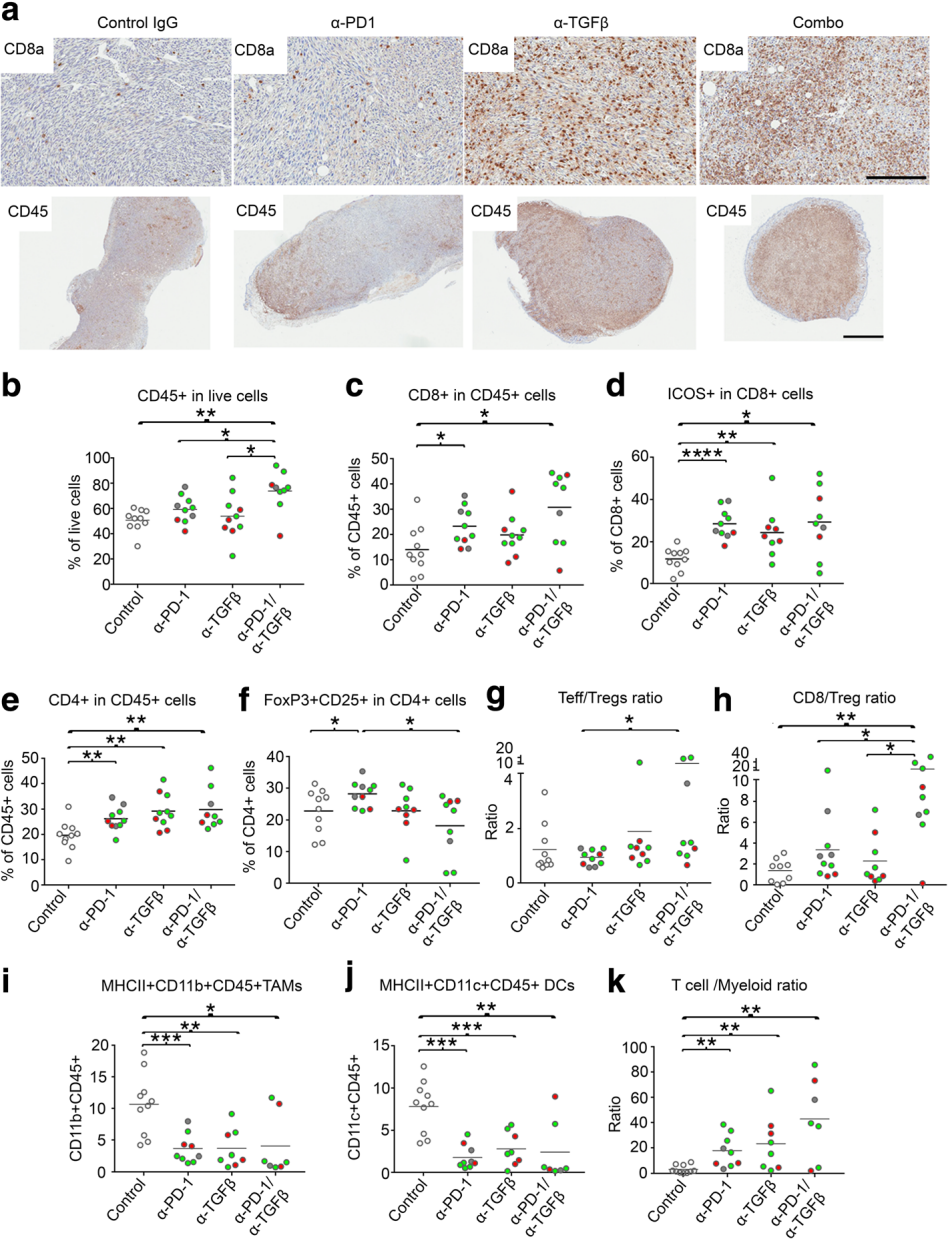
Immunophenotyping of CCK168 tumors in response to α-PD-1 and α-TGFβ therapy. **a**-**k**) CCK168 tumor cells were implanted sc into FVB mice according to Fig. 1b. After two drug doses on day 0 and 4, when some tumors began to show evidence of shrinkage, all tumors were harvested and analyzed by (**a**) immunohistochemistry or (**b**-**k**) multicolor flow cytometry. **a** Representative images of CD8a and CD45 immunohistochemistry for tumors from each of the four drug arms. Six to seven tumors were analyzed per drug arm per stain. Scale bar represents 50 uM. (**b**-**k**) Flow cytometry analysis shows increases in **b** CD45+ cells per live tumor cell, **c** CD8+ cytotoxic T cells per CD45+ immune cells and **d** increases in percentage of ICOS+ expressing CD8+ T cells. **e** α-PD-1 or α-TGFβ monotherapy elevates total CD4+ T cells with no additive effect. **f** α-TGFβ monotherapy neutralizes α-PD-1 induction of Tregs and, in combination therapy, reduces Treg levels to below baseline. **g** heterogeneous increase in CD4 + Th/CD4 + Treg ratio by α-TGFβ, **h** synergistic induction of CD8+/Treg ratios by α-PD-1 and α-TGFβ. The latter increased six to 40 fold in response to combinatorial therapy. (**i**) MHCII+CD11b + and (**j**) MHCII+CD11c + myeloid cells diminish as a percentage of total CD45 + cells following α-PD-1 or α-TGFβ therapy. **k** The ratio of mature T cells (CD4+ plus CD8+ cells) per CD11b myeloid cell (CD45 + Ly6G-CD11b + MHCII+) increases after combinatorial therapy. Flow cytometry data are representative of two to seven independent experiments for each cell type. * p < 0.05; ** p < 0.01; *** p < 0.001: Mann Whitney U test

We next sought to validate the possible mechanisms contributing to synergy between α-TGFβ and α-PD-1 antibodies in immunotherapy. IHC and flow cytometry analyses revealed that combinatorial therapy of CCK168 tumors with the two therapeutic antibodies caused a large increase in CD45+ leukocytes cells compared to either monotherapy (Fig. 4a, b). Tumor infiltration of cytotoxic CD8+ T cells was only marginally increased by α-PD-1 treatment, but the drug combination generated a robust CD8+ T cell influx (Fig. 4a, c). Furthermore, α-TGFβ or α-PD-1 monotherapy elevated the percentage of CD8+ T cells that express markers of late T cell differentiation and partial T cell exhaustion, ICOS and CTLA4 (Fig. 4d, Additional file 3: Figure S4), that have also been associated with active anti-tumor responses in human tumors [34]. However, even after combinatorial therapy, fewer than 50% of CD8+ T cells expressed these markers (Fig. 4d, Additional file 3: Figure S4). In contrast, the majority (70–90%) of CD4+ T cells, and specifically > 90% of CD4+ Tregs, expressed both ICOS and CTLA4 (Figs. S4, S5), suggesting that most intratumoral Tregs in CCK168 control tumors are in an activated state.

Importantly, α-TGFβ significantly reduced relative Treg levels (Fig. 4f), and variably enhanced CD4 + Th/Treg and CD8 + T/Treg ratios, in some cases up to forty fold, particularly in responding tumors (Fig. 4 g,h). In contrast to α-PD-1 monotherapy (Figs. 2a), α-TGFβ antibodies or the drug combination resulted in infiltration of total CD45+ and CD8a + T cells into the tumor core (Fig. 4a).

The myeloid MHCII+ antigen presenting cell population was substantially reduced by either drug given as a single agent, including reduced TAM and DC levels (Figs. 2 b, 4i-k, Additional file 3: Figure S6). Additionally, each monotherapy caused a slight but significant increase in the ratios of Ly6C^hi^/Ly6C^lo^ and CD11b^hi^CD11c^lo^/ CD11b^lo^CD11c^hi^ [28] TAMs (Fig. S6), suggesting a shift in the balance from immunosuppressive towards inflammatory TAMs, however, there was no synergistic effect of combinatorial therapy. Th17 T cell, natural killer cell and CD103+ DC numbers were all low and not significantly affected by drug treatment in CCK168 (not shown). This does not however exclude the possibility that these cell types play an important role in tumor responses. Migratory CD103+ DCs, for example, although a rare population within tumors, are essential for efficient antigen presentation within draining lymph nodes [28].

**Fig. 5:**
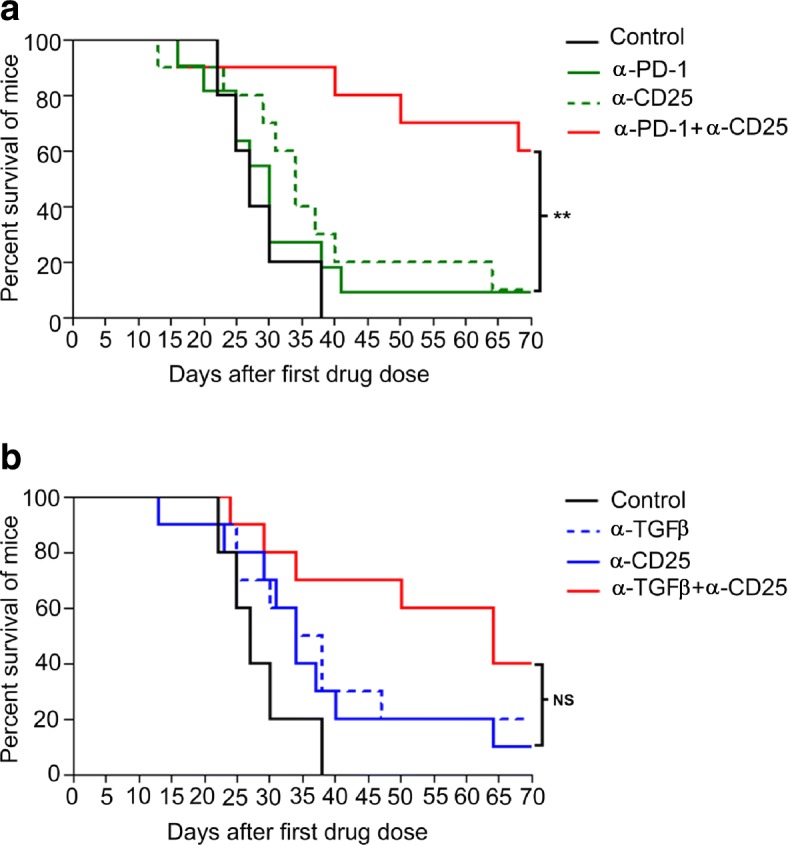
Treg depletion synergizes with α-PD-1 but not α-TGFβ in tumor regression and long term survival. CCK168 tumors were treated as in Fig. 1b, except that 24 h prior to the first therapeutic drug dose, mice received intraperitoneal injection of an IgG control or α-CD25+ cell-depleting antibody. a) and b) Kaplan Meier survival plots for CCK168 tumor-bearing mice using using 2000 mm3 tumor size as cut off for survival. *P* < 0.05 = *, *p* < 0.01 = ** Gehan-Breslow-Wilcoxon test

α-TGFβ antibodies thus have multiple effects on lymphocyte and myeloid cell molecular phenotypes, but the over-arching factors that correlate with synergism between α-TGFβ and α-PD-1 appear to be combinatorial increases in CD8+ cytotoxic T cells and reversal, by α-TGFβ, of the unfavorable CD4+ Treg/Th balance induced by α-PD-1 monotherapy. In order to demonstrate that Tregs are biologically active in CCK168 tumors, and limit tumor eradication by α-PD1, we depleted CD4 + CD25+ Tregs using an α-CD25 antibody engineered to optimize intra-tumoral ADCC of CD25+ Tregs [35]. Within 24 h of systemic α-CD25 administration, intra-tumoral Treg levels (CD4 + Foxp3+ and CD4 + CD25+) were reduced approximately 50% within CCK168 tumors (Additional file 3: Figure S7). Treg depletion alone, by α-CD25 treatment, had little effect on CCK168 tumor regression. Thus Tregs cannot be the only cellular target responsible for α-TGFβ-induced tumor regression in the absence of α-PD-1. Nevertheless, α-CD25 treatment exhibited significant synergy when combined with α-PD-1 (Fig. 5a), demonstrating that Tregs are functional and play a major role in restricting the anti-tumor activity of α-PD-1 in the CCK168 model. Combination of α-TGFβ therapy with α-CD25 Treg depletion shows a trend towards better anti-tumor activity than either treatment alone, but this effect did not reach statistical significance (Fig. 5b). Taken together, these data support the concept that α-TGFβ acts via both Treg-dependent and -independent mechanisms to elicit tumor rejection.

### α-PD-1 therapy enhances intra-tumoral TGFβ/pSmad3 signaling within CCK168 tumor cells

**Fig. 6:**
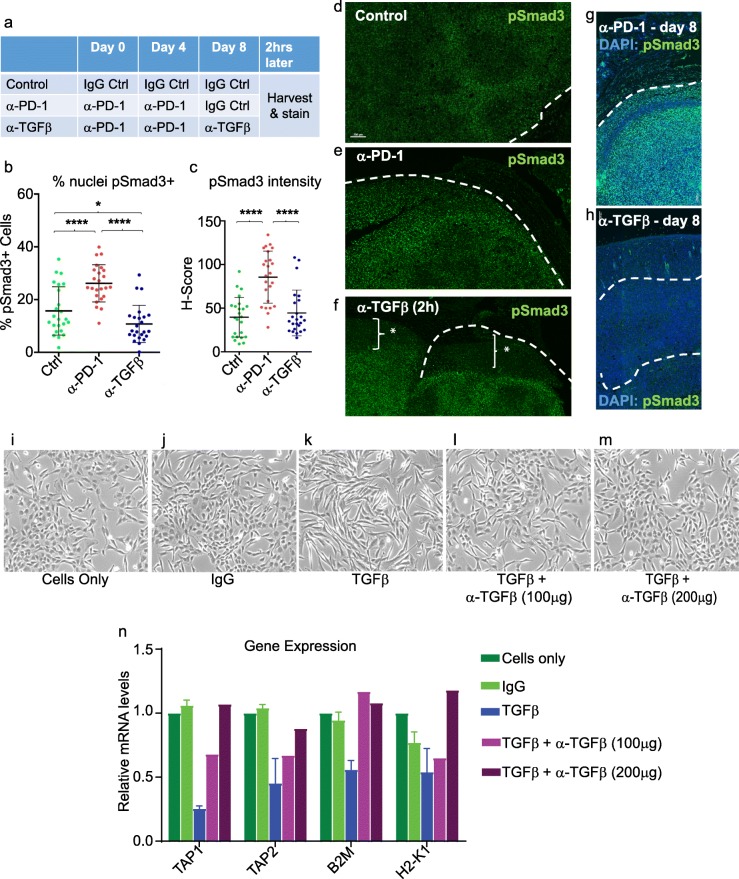
α-PD-1 therapy induces pSmad3 signaling in CCK168 tumors to enhance EMT and suppress gene expression of antigen presenting machinery. **a**-**f** CCK168 tumor-bearing mice were treated either with α-PD-1 monotherapy or IgG control antibodies on day 0 and 4. As indicated in (**a**), on day 8, the α-PD-1 treated group were randomly split into two further groups and treated with either control IgG or α-TGFβ monotherapy, and all tumors were harvested 2 h later for pSmad3 immunofluorescence staining. **b** and **c** quantification of the three arms of the experiment **b** as percentage of DAPI+ nuclei stained with pSmad3 and **c** intensity of pSmad3 staining per nucleus. **d**-**f** representative images of pSmad3 immunofluorescence staining. Note rim of tumor in α-TGFβ treated sample **f** shows dramatically reduced pSmad3 staining. **g**, **h** CCK168 tumor-bearing mice were treated with α-PD-1 or α-TGFβ on day 0 and day 4, and analyzed by pSmad3 immunofluorescence on day 8. **i**-**n** CCK168 cells grown in vitro were treated with TGFβ and/or α-TGFβ antibodies. I-m) phase contrast analysis shows reversible TGFβ-induced EMT. **n** RNA from cultures shown in (**l**-**m**) was extracted and subjected to qRT-PCR to quantify gene expression of components of the tumor cell antigen presentation machinery, *Mhc1*, *B2M* (β2-microglobulin), *Tap1* and *Tap2*. * = P < 0.05, **** = *P* < 0.0001; Unpaired two-tailed Student’s T test

To investigate which tumor cell types are responsive to TGFβ, we assessed pSmad3 immuno-fluorescence staining of tumor sections. Smad3 is a direct substrate for the transmembrane TGFβ type I receptor kinase and thus a marker of TGFβ pathway activation. In control tumors, we found high pSmad3 signaling in the majority of cells of the tumor parenchyma, with a heterogeneous staining pattern across each tumor, and variable expression between tumors (Fig. 6 a-d). In contrast, CD8+ cytotoxic T cells and CD163+ macrophages showed no pSmad3 staining (not shown). Unexpectedly, in two independent experiments (*n* = 3–5 tumors per arm per experiment), α-PD-1 therapy significantly increased pSmad3 levels over that observed in the IgG control group (Fig. 6 a-c, Additional 3: Figure S8). Both basal and α-PD1-induced pSmad3 were largely eliminated by α-TGFβ therapy (Fig. 6 b, c, and Additional file 3: Figure S8). As early as 2 h following ip injection of α-TGFβ antibodies (Fig. 6 f), pSmad3 staining was downregulated in the periphery but not within the core of the tumor parenchyma relative to that observed in IgG or α-PD-1-treated tumors (Fig. 6a-f). Eight days (two doses) following initiation of α-TGFβ monotherapy under the standard protocol (Fig. 1b), pSmad3 downregulation was extensive across the entire tumor, including the tumor core (Fig. 6g, h). The magnitude of this decrease was variable between tumors and generally associated with evidence of tumor regression.

### TGFβ induces EMT and reduces expression of tumor cell antigen presentation genes in CCK168 cells

In the mouse skin model of chemical carcinogenesis, TGFβ induces reversible EMT of tumor cells [8, 12] to promote tumor cell invasion and metastasis [11]. Indeed, even in CCK168 cells that are already spindle in phenotype, in vitro TGFβ treatment further stimulates EMT, generating larger more fibroblastoid cells, and this phenotypic switch is blocked by α-TGFβ (Fig. 6,i-m). In vivo*,* histological and pSmad3 immunofluorescence analysis of CCK168 tumors eight days following initiation of immunotherapy, suggests drug-induced changes in EMT (Fig. 6 and S9). All tumors were histologically heterogeneous, and both control and α-PD-1-treated tumors contained swathes of large, overtly fibroblastoid spindle tumor cells expressing high pSmad3 at both the tumor periphery and throughout the tumor (Figs. 6 e, Additional file 3: Figure S9). In α-TGFβ-treated tumors this overtly spindle phenotype was somewhat attenuated (Fig. S9), as previously observed in E4-derived SCCs after treatment with a small molecule TGFβR1 inhibitor [36]. Thus, aspects of reversible EMT observed in vitro*,* are also seen after αTGF-β therapy in vivo.

EMT involves large-scale changes in the global transcriptional program as cells transition from epithelial to fibroblastoid. To determine possible implications of these changes to innate tumor cell immunity*,* we investigated the effects of TGFβ or α-TGFβ on expression of genes encoding the antigen presentation machinery in CCK168 cells in vitro. Indeed, TGFβ treatment suppressed expression of *Mhc1, B2m, Tap1* and *Tap2*, whereas α-TGFβ treatment reversed these effects (Fig. 6n). Such molecular changes might contribute to the ability of cytotoxic T cells to recognize and destroy cancer antigen-expressing tumor cells.

### Heterogeneous responses of CCK168-derived tumors to α-TGFβ and α-PD-1 therapies.

**Fig. 7:**
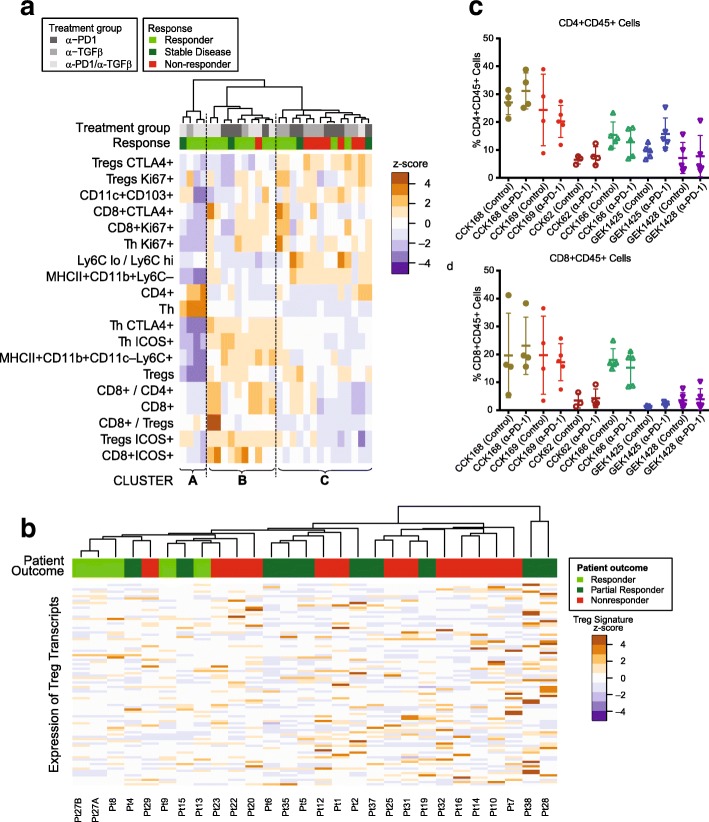
Heterogeneous mechanisms of tumor response to α-PD-1 and α-TGFβ therapy. **a** Unsupervised hierarchical clustering of indicated immune cell sub-populations in tumors after treatment with control IgG, α-PD-1, α-TGFβ, or combination therapy. Tumors were classified into responders (light green), stable disease (dark green) or non-responders (red) as described in Methods (colored horizontal bar). Responding mice tend to cluster into two groups that we label Responding Cluster A and Responding Cluster B, while non-responding mice tend to cluster into a third group (Cluster C). Mice in the two responding groups had significantly better outcomes than mice in the nonresponding cluster (*p* = 0.02, Fisher’s Exact test). Immune profiles of tumor infiltrates in Cluster B are characterized by high T cell levels and relatively low LyC6–macrophages (TAM2s), while those in Cluster A are characterized by low levels of all immune cell subtypes except CD4+ T cells, in particular high CD4 + FoxP3–CD25– (T helper) cells. **b** Unsupervised hierarchical cluster analysis undertaken according to expression of a Treg transcriptomic signature in pre-treatment human melanoma samples from patients treated with α-PD-1 (see Supplementary Methods). Transcriptomic data from pre-treatment melanoma samples [12] were subjected to unsupervised hierarchical cluster analysis based on gene transcripts whose expression correlates with FoxP3 expression in CD4+ cells. Human tumors responses were classified according to Hugo et al. 2016 [12] and the original tumor IDs are presented at the base of the figure. Samples from patients exhibiting a response, in particular CR, tended to cluster together (*P = 0.005,* Fisher’s Exact test) and to have reduced expression of FoxP3-associated genes. c,d) Each SCC line, in order of decreasing TML, CCK168, CCK169, CCK62, CCK166, GEK1425, GEK1428, was used to induced tumors in mice, and treated with α-PD-1 or control IgG on Day 0 and Day 4 (Fig. 1b). On day 8, tumors were harvested for flow cytometric analysis of immune cells. **c** CD4+ T cells per total CD45+, **d** CD8+ cytotoxic T cells per total CD45+

Of the six SCC lines studied, CCK168 is most sensitive to combinatorial immunotherapy but, even in this line, tumor responses are heterogeneous and only 60% of tumors achieve CR following α-PD-1/α-TGFβ combination therapy at the doses used. The extent of immune cell infiltration exhibited considerable heterogeneity between tumors derived from CCK168 cells (Fig. 4). We therefore sought to determine whether intratumoral leukocyte profiles could distinguish productive versus non-productive tumor responses. We undertook unsupervised hierarchical cluster analysis the CCK168 tumor immune cell profile data generated by flow cytometry following 8 days of IgG or drug treatment. This analysis revealed that responding tumors tended to segregate within one of two distinct clusters, Cluster A and Cluster B (Fig. 7a). Mice in these two “responding clusters” had significantly better outcomes than mice in Cluster C, the “nonresponding cluster” (*p* = 0.02, Fisher’s Exact test). Cluster B, the larger of the responder groups, was characterized by higher CD8+ T cell numbers, especially activated CD8 + ICOS+ and CD8 + CTLA4+ T cells, higher inflammatory Ly6C^hi^ TAM numbers, but lower levels of Ly6C^lo^ TAMs. The second, smaller responding cluster, Cluster A, had relatively low levels of all immune cell types except for highly prevalent CD4+ T cells (CD4 + FoxP3-CD25-). The third cluster of predominantly non-responding tumors, Cluster C, was characterized by lower levels of CD8+ cells and Ly6C^hi^ TAMs, but higher levels of CTLA4 + Tregs, Ly6C^lo^ TAMs and a high Ly6C^lo^/Ly6C^hi^ TAM ratio, compared to responding Clusters A and B (Fig. 7a). Thus it appears that there are two classes of responsive CCK168 SCCs, those with high and those with low CD8+ cytotoxic T cell infiltration, with the latter class having a higher CD4+ Th cell content that may be particularly responsive to α-TGFβ.

To address the translational relevance of Tregs as a biomarker of resistance to α-PD-1 therapy, we investigated the link between pretreatment intratumoral activated Treg levels and clinical response to α-PD-1 in melanoma patients. We interrogated transcriptomic data from a study of 38 pembrolizumab (α-PD-1) pretreatment melanoma tumors for which clinical outcomes data were available (21 responding versus 17 non-responding) [13]. Unsupervised hierarchical cluster analysis based on a Foxp3-associated transcriptional CD4+ T cell signature [37] showed that tumors from patients with subsequent CR on clinical follow up clustered together based on lower expression of this Treg signature (Fig. 7b, *p = 0.005*).

### CD4+ T cell content distinguishes responding from non-responding SCC tumor lines

Having established the heterogeneity of tumor responses to therapy within one tumor line, CCK168, we next addressed which determinants across the SCC panel, other than mutation load, might also associate with tumor rejection after α-TGFβ or α-TGFβ plus α-PD-1 combination therapy. All six lines expressed comparable levels of total TGFβ1 and TGFβ2 proteins, with little expression of TGFβ3 in vitro, as determined by ELISA (data not shown). Moreover, each line showed sensitivity to TGFβ in vitro as demonstrated by activation of pSmad2/3 in response to TGFβ (data not shown).

Since no obvious in vitro characteristic distinguished the six SCC lines from one another, and the only parameter that appears to associate with tumor response to immunotherapy was TML, we asked whether distinct immune cell subsets within pre-treatment or α-PD-1-treated tumors distinguish responsive from non-responsive SCC lines. IHC analysis of intra-tumoral T and myeloid cell populations (Fig. S10) distinguished two tumor classes according to infiltrating CD45+ leukocyte and CD3+ T lymphocyte content. GEMM-derived tumors, GEK1425 and GEK1428, and the non-responsive carcinogen-induced carcinoma, CCK62, showed very little infiltration by T cells (CD3+ or CD8a+) (Fig. S10), and this was largely unaltered by therapy. Conversely, levels of CD3+ and CD8a + T cells were higher in the responsive lines CCK168 (Figs. 2a, 4a, Additional file 3: FigureS10) and CCK169 SCCs (Fig. S10), and further induced by combination therapy. Paradoxically, CCK166 tumors that are resistant to immunotherapy and have the lowest TML of the chemically-induced SCC subset, nevertheless had CD45+, CD3+ and CD8+ T cell levels comparable to those of the two high TML, drug-responsive SCC lines. CD45+, CD3+ and CD8+ cell levels also increased in CCK166 in response to combinatorial therapy (Fig. S10). This demonstrates the lack of correlation between tumor immune cell infiltrate and anti-tumor response, even in this small panel of SCCs.

In order to consolidate and extend this finding, we compared levels of intra-tumoral CD45+ leukocytes, CD8+ cytotoxic T cells, total CD4+ T cells, Foxp3+ Tregs, and myeloid cells by multi-color flow cytometry (Fig. 7 c,d). The only pre-treatment parameter we found to associate with response to combination therapy was total CD4+ T cell content. Control-treated CCK168 and CCK169 tumors, had a significantly greater CD4+ T cell content than tumors of the unresponsive tumor lines (Fig. 7c, *p = 0.001*). This observation is consistent with the finding of a subcategory of CCK168 drug-responsive tumors marked by high CD4+ T cell levels (Fig. 7a), that may be an important determinant of synergy between α-PD-1 and α-TGFβ in SCCs.

## Discussion

In the current study, we present a novel panel of chemically-induced and GEMM-derived cSCC tumor lines and show that only those with high TML respond to immunotherapy. We also make the novel finding that in such SCCs, α-PD-1 therapy not only induces cytotoxic T cell activity, but also induces a competing TGFβ-driven immunosuppressive program that restrains its anti-tumor activity. We demonstrate that α-PD-1 therapy results in skewing of the CD4+ Teff/Treg balance in favor of immunosuppressive Tregs, and these Tregs functionally limit the anti-tumor activity of α-PD-1 in CCK168 SCCs. We also demonstrate that α-PD-1 therapy enhances TGFβ-Smad3 signaling within tumor cells, that contributes to EMT. α-TGFβ monotherapy is consequently more efficacious (20% CR) than α-PD-1 monotherapy (< 3% CR), as it targets both Tregs and tumor cell EMT while also stimulating the effector arms of both the innate and adaptive immune systems. Importantly, the two drugs synergize when used in combination to provide 60% CR in established CCK168 tumors.

Recent analyses of pre-treatment immune-excluded urothelial cancers and human melanomas, revealed enrichment for a transcriptomic signature of TGFβ signaling [16] and TGFβ-driven mechanisms, such as EMT and immunosuppression [13], in patients with poor clinical outcomes following subsequent blockade of the PD-1/PDL-1 axis. Our current findings are not inconsistent with these clinical studies, but go farther to show that α-PD-1 monotherapy itself promotes increased tumor cell TGFβ signaling and elevates the immunosuppressive Treg/Th balance, consequently limiting the efficacy of this checkpoint blockade drug. This unexpected activity of α-PD-1 in inducing the TGFβ signaling axis may contribute to the recently described clinical phenomenon of α-PD-1 super-progressors following checkpoint blockade therapy that has been observed in several cancer types [3–5].

Mariathasan et al. [16] and Tauriello et al. [17] recently demonstrated that stromal cancer-associated fibroblasts (CAFs) are the major cell source and responders to TGFβ signaling in colon and urothelial carcinomas, respectively. These studies suggest that CAFs create an immunosuppressive barrier preventing penetration of T cells into the tumor proper. However, in the immune-infiltrated CCK168 model, CAFs do not appear to play such a role since most non-immune cells are tumor cells rather than CAFs, as shown by expression of cytokeratin K8 and K18 (not shown). Nevertheless, by virtue of TGFβ-induced EMT, tumor cells themselves may acquire a CAF-like phenotype and fibroblastic immunosuppressive functions. Under the influence of α-PD-1, elevated pSmad3 may drive EMT towards a more extreme myofibroblast phenotype, reduced expression of antigen presentation machinery and changes in the secreted cytokine and extracellular matrix profiles that suppress tumor immune recognition. Indeed, histological analysis of CCK168 tumors suggests a more overt spindle phenotype after α-PD-1 therapy and reversal of this effect after α-TGFβ therapy, and in vitro data demonstrate reversible activity of TGFβ in induction of CCK168 EMT and suppression of expression of components of the antigen presentation machinery.

The mechanism of α-PD-1-elevated pSmad3 signaling in tumor cells is unlikely direct, since CCK168 cells express no PD-1 and only low levels of PD-L1 and PD-L2 (data not shown). In the transgenic adenocarcinoma of mouse prostate (TRAMP) model, activated effector CD4+ T cells have been reported to release active TGFβ1 [38]. Moreover, according to conventional wisdom, Tregs are a major source of TGFβ. α-PD-1 therapy may therefore enhance TGFβ activity through its activation of CD4+ T cells, both Tregs and Th1 cells. TAMs and/or DCs may also release and/or activate TGFβ that drives Treg differentiation [39]. Notably, PD-1 is expressed on mouse and human TAMs [40], but the consequence of PD-1 blockade on secretion or activation of TGFβ has not been investigated. The elevated intratumoral Treg/Th balance that we observe following α-PD-1 monotherapy may be due to prevention of Treg anergy by blockade of PD-1 expressed on Treg cells. Alternatively, α-PD-1 may increase Treg differentiation indirectly, in response to active TGFβ released from other cellular or extracellular sources, such as activated CD4+ Th cells [38] or immunosuppressive myeloid cells. Precedent for the direct activation of Tregs by α-PD1 comes from studies in non-neoplastic disease models, such as those of infection and allergy, where PD-1 plays a role in inducing Treg anergy [41, 42].

High pre-treatment tumor CD4+ T cell content is the only parameter, apart from high TML, that associates with immunotherapy responses in two of the panel of six SCC tumor lines studied here. CD4+ T cells, unlike the cytotoxic T cell lineage, show considerable plasticity in differentiation, such that Tregs are capable of re-differentiation towards an inflammatory Th phenotype [43, 44] that may be modulated by α-TGFβ immunotherapy. α-TGFβ or a combination of α-PD-1 with α-TGFβ might therefore be indicated for that category of patients with a high intratumoral CD4+ T cell or Treg content. Such an intratumoral immune cell profile has been reported for SCC-HN [45], which also bear high TMLs [6] and have been reported to undergo super-progression in response to a-PDL-1 therapy [5].

Additive, synergistic and redundant anti-tumor interactions between TGFβ signaling and the PD-1/PD-L1 axis [18–22] may be influenced by host genetic background [46–48]. Characterization of such interactions and development of predictive biomarkers for response to α-PD-1/α-TGFβ therapy is therefore of high priority. The panel of syngeneic carcinomas presented here, comprising cell lines with a range of mutation burdens and immune cell profiles, all on the same FVB genetic background, and driven by the most common oncogenic signaling pathway, Kras, provides additional opportunities to investigate the disparate mechanisms of innate and acquired resistance to immunotherapies that may be encountered in the clinic. Notably, the potent and specific TGFβ blocking antibodies used in our studies have entered clinical trial in combination with α-PD-1 for oncology (NCT02947165), and additional drugs that impact this pathway [15] may also prove effective in combinatorial immunotherapy with checkpoint blockade drugs.

## Conclusions

We show that α-PD-1 not only initiates a tumor rejection program, but can induce a competing TGFβ-driven immuno-suppressive program in SCCs. α-PD-1 monotherapy skews the CD4 + T cell balance in favor of immunosuppressive Tregs, and elevate pSmad3 signaling within tumor cells, features that are blocked by α-TGFβ therapy. In SCCs, TGFβ blockade acts through both tumor cell autonomous and immune-mediated mechanisms to stimulate anti-tumor immunity and alleviate α-PD-1 resistance. This study forms the basis for a clinical trial of α-TGFβ/α-PD-1 combination therapy (NCT02947165).

## Additional files

Additional file 1: Supplementary Methods. (DOCX 9 kb)

Additional file 2: **Table S1.** Mutation counts of tumor cell lines. (XLSX 9 kb)

Additional file 3: **Figure S1.** Growth responses of CCK62 and GEK1428 to immunotherapy. **Figure S2.** CCK168 responses to α-PD-1 and/or α-TGFβ. **Figure S3.** α-TGFβ and α-TGFβ/α-PD-1 combination therapy elicit long-term tumor immunity to Kras-driven tumors. **Figure S4.** Gating strategy for T cell flow cytometry and analysis of differentiation and proliferation markers. **Figure S5.** Gating strategy for Th versus Treg cell flow cytometry and analysis of differentiation and proliferation markers. **Figure S6.** Gating strategy for myeloid cell flow cytometry and analysis of macrophage and dendritic cell markers. **Figure S7.** Depletion of Tregs 24 hours after anti-CD25 antibody treatment. **Figure S8.** α-PD-1 induces pSmad3 in CCK168 cells. **Figure S9.** CD3 T cell staining and histology of tumors after treatment with each of the four drug arms as indicated. **Figure S10.** IHC analysis of immune infiltrates in tumors. (PDF 9660 kb)

Additional file 4: **Table S2.** List of all nonsynonymous coding mutations in six tumor cell lines. (XLSX 84 kb)
