# Supplementary material for: α-PD-1 therapy elevates Treg/Th balance and increases tumor cell pSmad3 that are both targeted by α-TGFβ antibody to promote durable rejection and immunity in squamous cell carcinomas
Source: J Immunother Cancer. 2019 Mar 4;7:62. doi: 10.1186/s40425-018-0493-9 (PMC6399967; doi:10.1186/s40425-018-0493-9)
Supplement: Supplementary file 1 — Supplementary Methods. (DOCX 9 kb) [file 40425_2018_493_MOESM1_ESM.docx]

**Supplementary Methods:**

**Mouse experiments**

FVB/NJ mice (6-week old females) were purchased from the JAX labs and acclimatized to the UCSF Laboratory Animal Research Center facility for one week, were used for experiments. All animal procedures adhered to NIH Guidelines for the Care and Use of Laboratory Animals. Procedures were undertaken under authorization of the UCSF Institutional Animal Care and Use Committee in an AAALAC approved facility.

**Generation of SCC cell lines**

Chemically-induced cutaneous SCCs were generated on wild type or *Hras-/-* (1) mice of the FVB/NJ strain using a standard DMBA/TPA protocol (2). cSCC appear between 25-45 weeks following a single topical treatment with DMBA (25μg in 200 μl acetone applied to previously shaved dorsal skin) followed by twenty weeks of biweekly TPA application (200μl per treatment of a 10^-4^ M solution in acetone).

GEMM-derived cSCCs were generated by intercrossing FVB mice carrying *Kras^LSL-G12D^*, (3) and *Lgr5-CreER* (4) alleles. Activation of Cre within Lgr5-expressing keratinocytes was induced by a single topical application of 4-hydroxytamoxifen (200 ul of 25 mg/ml in 100% ethanol), causing replacement of *Kras* with an activated *KrasG12D* allele by Cre-induced recombination of *Kras^LSL-G12D^*. Papillomas arose 6-8 weeks after induction of a full thickness wound. Papillomas >5mm were excised and allowed to grow back, after which they converted to SCCs. GEMM-derived cSCC developed 12-15 weeks following papilloma resection.

Primary cSCCs were removed from freshly euthanized mice and, under sterile conditions, chopped vigorously into small pieces before plating into cell culture with DMEM supplemented with 10% fetal calf serum, non-essential amino acids and penicillin/streptomycin. Attached cells were expanded for 3-4 weeks before stocks were frozen down, and all experiments were undertaken on cells at ≤ four passages. Tumor lines were validated by whole exome sequencing and confirmed to be mycoplasma-free.

**In vivo experiments**

After expansion in DMEM plus 10% FCS, 1.5 x 10^4^ tumor cells were injected subcutaneously and unilaterally into the dorsal flank of 7 to 8 week old mice. 14 days later, when tumors measured approximately 5 mm diameter, mice were injected ip with three doses of therapeutic antibodies, administered at four day intervals. Antibodies used were rat α-PD-1 IgG2a antibody (RMP1-14, BioXCell) or its rat isotype control (2A3) at 250μg per dose, and humanized α-pan-TGFβ1,2,3 IgG2a (XPA-42-068.1, Xoma Corp), humanized anti-TGFβ1,2 IgG2a (XPA-42-089, Xoma Corp) or humanized isotype control antibody (α-KLH2, Xoma Corp) each at 200μg per dose (5).

**DNA extraction & whole exome sequencing**

Cell line samples were submitted to Otogenetics Corporation (Atlanta, GA USA) for mouse exome capture and sequencing. Isolation of genomic DNA (gDNA), library preparation for sequencing, and exome sequencing were performed by Otogenetics. gDNA was isolated from tumor cell lines as well as from two strain-matched mouse tails using DNeasy Blood & Tissue kit (Qiagen #69506). Briefly, gDNA was subjected to agarose gel and OD ratio tests to confirm the purity and concentration prior to Bioruptor (Diagenode, Inc., Denville, NJ USA) fragmentation. Fragmented gDNAs were tested for size distribution and concentration using an Agilent Tapestation 2200 and Nanodrop. Illumina libraries were made from qualified fragmented gDNA using SPRI works HT Reagent Kit (Beckman Coulter, Inc. Indianapolis, IN USA, catalog# B06938) and the resulting libraries were subjected to exome enrichment using SureSelectXT Mouse All Exon (Agilent Technologies, Wilmington, DE USA, catalog# 5190-4641) following manufacturer’s instructions. Enriched libraries were tested for enrichment by qPCR and for size distribution and concentration by an Agilent Bioanalyzer 2100. The samples were then sequenced on an Illumina HiSeq2500 using Rapid v2 SBS chemistry which generated paired-end reads of 106 nucleotides (nt).

**Sequence alignment, processing and quality control**

Reads were mapped to the GRCm38/mm10 version of the *Mus musculus* genome using BWA (version 0.7.12) (6) with default parameters. The Picard MarkDuplicated module was used to remove duplicates from the data (version 1.119; [http://broadinstitute.github.io/picard](http://broadinstitute.github.io/picard/)). The Genome Analysis Tool Kit (GATK-Lite) toolkit (version 2.3-9) module IndelRealigner and BaseRecalibrator were used to preprocess the alignments. During base quality recalibration, dbSNP variants were used as known sites, according to GATK Best Practices recommendations (7, 8). Finally, alignment and coverage metrics were collected using Picard. We sequenced an average of 47 million unique reads per sample. Targeted bases were sequenced to a mean depth of 62, and more than 88% of targeted bases were sequenced to 20× coverage or greater.

**Identification of SNVs and annotation**

SNVs were called using somatic variant detection program, MuTect (version 1.1.7) (9). Each tumor was called against a tail from a strain-matched mouse. Calls were filtered against a database of known *Mus musculus* germline SNPs available at [ftp.ncbi.nih.gov/snp/organisms/mouse_10090/](ftp://ftp.ncbi.nih.gov/snp/organisms/mouse_10090/) VCF/genotype, as well as against both normal tails from this experiment. Results were further filtered to calls with a minimum read depth of 10 at the locus for both tumor and matched normal, and to calls where at least one alternate read had a mapping quality score of 60 or higher. Variants were annotated using Annovar (downloaded on 2/4/2016) (10), and these annotations were used as the basis for assessing exonic variants as synonymous, nonsynonymous, stopgain, or stoploss.

**CD8+ cell depletion study:** CCK168 tumor growth analysis was undertaken for control IgG versus anti-PD-1/anti-TGFβ combo therapy (Fig.1b) except that animals were treated or not with a CD8 cell depleting anti-CD8a antibody (200μg per mice, UCSF Monoclonal Antibody Core, #53.6.72, Lot MC10148), administrated intraperitoneally 24 hours prior to each therapeutic drug dose.

**CD25+ cell depletion study:** CCK168 tumor growth analysis was undertaken for control IgG versus anti-PD-1/anti-TGFβ combo therapy (Fig.1b) except that animals were treated with a CD25+ cell depleting antibody (200μg per mice (11) or matched IgG control, administrated once, intraperitoneally, 24 hours prior to the first therapeutic drug dose.

**Flow Cytometry Analysis**

Flow analysis of each tumor cell parameter was undertaken in two to four independent experiments with seven to 10 tumors per experimental arm. Experiments were independently undertaken by three separate investigator teams (DSM, MLB and MB; ED and BL; and RP and MDR) using different FACS analysis panels, but with similar outcomes. Tumor cells were implanted in mice and treated with drugs as in Fig. 1b, except that only two doses of drug were administered on day 0 and day 4. When some mice showed evidence of initiation of an anti-tumor response (7-8 days after the first drug dose), all mice from that experiment were euthanized, and tumors harvested. Tumors were chopped vigorously by razor blade, and digested in 3 mL of RPMI-1640 medium containing 100 U/ml Collagenase A (Worthington), 500 U/ml Collagenase D (Worthington) and 200 μg/ml Dnase I (Roche) for 30 minutes at 37°C. Cell suspension was filtered through a 40 μm cell strainer.

Cell viability was assessed using fixable Live/Dead dye (Biolegend, # 423105). For surface staining, cells were incubated on ice with antibodies in PBS + 2% fetal calf serum and 2 mM EDTA for 30 minutes. Antibodies used were: CD45-1 (Alexa-Fluor 700, Biolegend #110724 A20, 1:500); CD4 (BV650-A, Biolegend #100546, RM4-5, 1:500); CD8 (BV605-A; Biolegend #100743, 53-6-7, 1:500); FoxP3 (eFluor 450, eBioscience# 48-5773-82, FJK-16s, 1:100); CD25 (eFluor780, eBioscience #47-0251-82, PC61.5, 1:100); Ki-67 (PE-Cy7 (yg), BD Biosciences #561283, B56, 1:100); ICOS (FITC-A, eBioscience #11-9949-80, C398.4A, 1:200); CTLA4 (PE(yg), BD Bioscience #553720, UC10-4F10-11, 1:100); Ly6G (PE-Cy7, Biolegend # 127645, 1A.8, 1:100); CD11b (APC-eFluor780, eBioscience#47-0112-82, 47-0112-82, 1:100); CD45 (Alexa-Fluor 700, Biolegend #110724, A20, 1:200); F4/80 (BV785, Biolegend#123109, BM8, 1:100) CD11c (BV650; Biolegend#117339, N418, 1:100); Ly6C (BV605, Biolegend#128035, HK1.4, 1:100); MHC II (PB/e450,eBioscience#48-5321-80, M5/114.15.2, 1:500); Brilliant Violet (BV) 605^TM^ anti-mouse CD45 (Biolegend #103139, 30-F11 1:500 dilution); PerCP/Cy5.5 anti mouse CD45 (Biolegend #103131. 30-F11 1:500); BV 785^TM^ anti-mouse/human CD45R/B220 (Biolegend #103245, RA3-6B2, 1:1’000); PE/Cy7 anti-mouse CD4 (Biolegend #100421, GK1.5, 1:500); PerCP/Cy5.5 anti-mouse CD8a (Biolegend 100733, 53-6.7, 1:500); BV 605^TM^ anti-mouse/human CD11b (Biolegend #101237, M1/70, 1:2000); PE/Cy7 anti-mouse CD11c (Biolegend #117317, N418, 1:500); FITC anti-mouse CD25 (Biolegend #102005, PC61,1:200); BV 711^TM^ anti-rat CD90/mouse CD90.1 (Thy-1.1 (Biolegend #202539, OX-70, 1:500); APC anti-mouse CD103 (Biolegend #121413, 2E7, 1:500); anti-mouse/rat Foxp3 eFluor® 450 (eBioscience #48-5773-80, FKJ-16s, 1:100); FITC anti-mouse F4/80 (Biolegend #123107, BM8, 1:500) and BV 421^TM^ anti-mouse I-A/I-E (Biolegend #107631, M5/114.15.2, 1:500). Intracellular FoxP3 stain was performed using the FOXP3 fix/perm buffer set (Biolegend #421403) according to the manufacturer’s protocol. Unless an intracellular stain was performed, stained cells were fixed with 2% paraformaldehyde for 10 minutes at 25°C. All flow cytometry was performed on a BD Fortessa flow cytometer. Analysis of flow cytometry data was done using Flowjo (Treestar). Representative flow analyses are shown in Supplementary figures.

**Classification of responders versus progressive disease:**

For tumor growth analysis, individual tumors were classified as complete responders (CR), partial responders (PR), or progressive disease (PD), according to their growth characteristics (CR, tumor eradication with no regrowth; PR, tumor shrinkage ≥30%; PD, no effect of drugs compared to control IgG).

For flow cytometry analysis undertaken seven to eight days after the first drug treatment, tumors were classified as responders (PR), stable disease (SD) or progressive disease (PD) according to their individual growth trajectories compared to the mean tumor volume of control tumors (for example see Fig.S6). PD was taken as consistently increasing in tumor volume above or around the mean of control tumors. PR was considered a decrease in tumor volume of >20% comparing each tumor longitudinally, or tumors with persistently smaller volumes (<50% of the mean volume of the controls) from the beginning of drug treatment. Exceptions to this were two mice that showed rapid PD until a dramatic decrease in volume in the last 24 hours of tumor growth before harvest, which were considered to be responders. SD tumors showed only a moderate increase in size with low variation and a size consistently below the mean of the controls.

**Unsupervised hierarchical clustering and heatmaps.** Unsupervised hierarchical clustering was performed in R, and the resulting heatmaps were generated using gplots. For the immune profile heatmap, raw data was taken to be cells expressing the specified markers as a percent of a parent population, as follows. CD4+, CD8+, and myeloid populations were measured as percent of CD45+ cells. CD4+Tregs and CD4+Th were measured as percent of total CD4+ cells. Ki67+, CTLA4+, ICOS+ measurements were a percent of the relevant population (e.g., CD8+Ki67+ is percent of CD8+). Input data matrices were scaled to compute a normalized z-score for each attribute, and samples were clustered using Ward’s method.

**Analysis of Treg signature in patients.** Patient RNAseq FPKM data and individual responses to α-PD-1 therapy were obtained from the GEO database, accession number GSE78220 (12). Treg signature was defined by a previously-published 100-gene module of RNA transcripts that correlated with expression of *FoxP3* within CD4+ T cells (13). FPKM data was subset to transcripts associated with genes in this FoxP3 module, and unsupervised hierarchical clustering was performed as described above. The responding patient cluster was defined as patients 4, 8, 9, 13, 15, 22, 23, 27A, 27B, and 29, and Fisher’s exact text was performed to calculate the p-value between responses of patients who were in *versus* those not in this responding cluster.

**Immunohistochemistry (IHC).** 4% paraformaldehyde or Zinc-fixed tissue samples in 70% ethanol were sent for processing and immunostaining at the UC Davis Center for Genomic Pathology Laboratory, Davis, CA. All IHC was performed manually without the use of automated immunostainers. The same antigen retrieval method was used for all IHC and was performed using a Decloaking Chamber (Biocare Medical, Concord, CA) with 10 mM citrate buffer at pH 6.0, 125°C and pressure to 15 p.s.i. The total time slides were in the chamber was 45 min. Both the CD8a (eBiosciences, Catalog # 14-0808, 1:400 dilution) and CD45 (BD Pharmingen, Catalog # 01111D, 1:400 dilution) primary antibodies were made in rat. Incubations with primary antibodies were performed at room temperature overnight in a humidified chamber. Normal goat serum was used for blocking. Biotinylated goat anti-rat (1:500) was the secondary antibody with a Vectastain ABC Kit Elite and a Peroxidase Substrate Kit DAB (Vector Labs, Burlingame, CA) used for amplification and visualization of signal, respectively. Tissues known to contain each assessed antigen were used as positive controls. Antibody deletion controls were used for every assessed antigen to confirm specific staining. Tissues stained with chromogenic IHC were visualized and scanned using an Aperio AT2 ScanScope (Leica Biosystems), and digital images were viewed using the ImageScope application (Leica Biosystems. Antibodies used for IHC were: anti-CD3 (clone SP7, Abcam), anti-CD8a (4SM15, eBioscience), anti-CD45 (clone EP322Y, Novus Biologicals) and anti-CD163 ().

**Quantitative Immunofluorescence Analysis:**

IHC with fluorescent-labelled probes and DAPI counterstain was performed on paraffin embedded tissue sections as described above. Tyramide signal amplification (TSA)-based fluorescence color visualization (14) was undertaken to analyze pSMAD3 levels and its distributions in tumor tissues (anti-pSMAD3; Abcam Ab52903). TSA stained tissues were visualized with a Vectra automated quantitative pathology imaging system (Perkin Elmer), and images were analyzed for nuclear localization of pSMAD3 with inForm cell analysis software (Perkin Elmer). H-Score was calculated as 3 x percentage of strongly staining nuclei + 2 x percentage of moderately staining nuclei + percentage of weakly staining nuclei. At least 8 areas in each tumor were analyzed for H-Score.

**Statistics:** Statistical analyses were performed using GraphPad Prism software (version 7.03; GraphPad Software), unless otherwise stated. For comparisons between the means of two variables, we used the Mann-Whitney U test. Comparison of survival data between the groups was undertaken using the Gehan-Breslow-Wilcoxon test after Kaplan-Meier analysis. A P value of <0.05 was taken as statistically significant.

1. To MD, Rosario RD, Westcott PM, Banta KL, Balmain A. Interactions between wild-type and mutant Ras genes in lung and skin carcinogenesis. Oncogene. 2013;32:4028-33.

2. Cui W, Fowlis DJ, Bryson S, Duffie E, Ireland H, Balmain A, et al. TGFbeta1 inhibits the formation of benign skin tumors, but enhances progression to invasive spindle carcinomas in transgenic mice. Cell. 1996;86:531-42.

3. Jackson EL, Willis N, Mercer K, Bronson RT, Crowley D, Montoya R, et al. Analysis of lung tumor initiation and progression using conditional expression of oncogenic K-ras. Genes Dev. 2001;15:3243-8.

4. Schepers AG, Snippert HJ, Stange DE, van den Born M, van Es JH, van de Wetering M, et al. Lineage tracing reveals Lgr5+ stem cell activity in mouse intestinal adenomas. Science. 2012;337:730-5.

5. Bedinger D, Lao L, Khan S, Lee S, Takeuchi T, Mirza AM. Development and characterization of human monoclonal antibodies that neutralize multiple TGFbeta isoforms. MAbs. 2016;8:389-404.

6. Li H, Durbin R. Fast and accurate short read alignment with Burrows-Wheeler transform. Bioinformatics. 2009;25:1754-60.

7. DePristo MA, Banks E, Poplin R, Garimella KV, Maguire JR, Hartl C, et al. A framework for variation discovery and genotyping using next-generation DNA sequencing data. Nat Genet. 2011;43:491-8.

8. McKenna A, Hanna M, Banks E, Sivachenko A, Cibulskis K, Kernytsky A, et al. The Genome Analysis Toolkit: a MapReduce framework for analyzing next-generation DNA sequencing data. Genome Res. 2010;20:1297-303.

9. Cibulskis K, Lawrence MS, Carter SL, Sivachenko A, Jaffe D, Sougnez C, et al. Sensitive detection of somatic point mutations in impure and heterogeneous cancer samples. Nat Biotechnol. 2013;31:213-9.

10. Wang K, Li M, Hakonarson H. ANNOVAR: functional annotation of genetic variants from high-throughput sequencing data. Nucleic acids research. 2010;38:e164.

11. Arce Vargas F, Furness AJS, Solomon I, Joshi K, Mekkaoui L, Lesko MH, et al. Fc-Optimized Anti-CD25 Depletes Tumor-Infiltrating Regulatory T Cells and Synergizes with PD-1 Blockade to Eradicate Established Tumors. Immunity. 2017;46:577-86.

12. Hugo W, Zaretsky JM, Sun L, Song C, Moreno BH, Hu-Lieskovan S, et al. Genomic and Transcriptomic Features of Response to Anti-PD-1 Therapy in Metastatic Melanoma. Cell. 2016;165:35-44.

13. Linsley PS, Chaussabel D, Speake C. The Relationship of Immune Cell Signatures to Patient Survival Varies within and between Tumor Types. PLoS One. 2015;10:e0138726.

14. Stack EC, Wang C, Roman KA, Hoyt CC. Multiplexed immunohistochemistry, imaging, and quantitation: a review, with an assessment of Tyramide signal amplification, multispectral imaging and multiplex analysis. Methods. 2014;70:46-58.
