# Supplementary material for: α-PD-1 therapy elevates Treg/Th balance and increases tumor cell pSmad3 that are both targeted by α-TGFβ antibody to promote durable rejection and immunity in squamous cell carcinomas
Source: J Immunother Cancer. 2019 Mar 4;7:62. doi: 10.1186/s40425-018-0493-9 (PMC6399967; doi:10.1186/s40425-018-0493-9)
Supplement: Supplementary file 3 — Figure S1. Growth responses of CCK62 and GEK1428 to immunotherapy. Figure S2. CCK168 responses to α-PD-1 and/or α-TGFβ. Figure S3. α-TGFβ and α-TGFβ/α-PD-1 combination therapy elicit long-term tumor immunity to Kras-driven tumors. Figure S4. Gating strategy for T cell flow cytometry and analysis of differentiation and proliferation markers. Figure S5. Gating strategy for Th versus Treg cell flow cytometry and analysis of differentiation and proliferation markers. Figure S6. Gating strategy for myeloid cell flow cytometry and analysis of macrophage and dendritic cell markers. Figure S7. Depletion of Tregs 24 hours after anti-CD25 antibody treatment. Figure S8. α-PD-1 induces pSmad3 in CCK168 cells. Figure S9. CD3 T cell staining and histology of tumors after treatment with each of the four drug arms as indicated. Figure S10. IHC analysis of immune infiltrates in tumors. (PDF 9660 kb) [file 40425_2018_493_MOESM3_ESM.pdf]

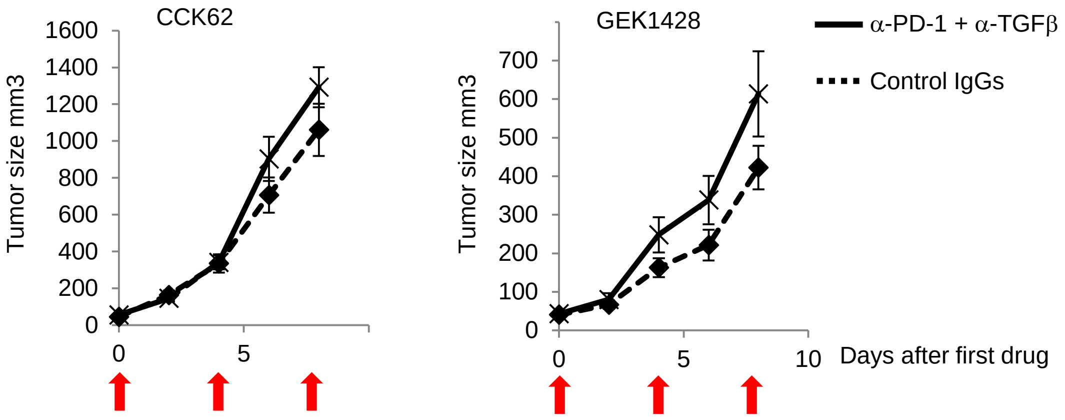


**Figure S1. Growth responses of CCK62 and GEK1428 to immunotherapy.** Tumors were generated according to the scheme in Fig 1b. Average tumor sizes of CCK62, CCK169 and GEK1428 in vivo, with and without anti-PD-1 and anti-TGFbeta were measured at least every other day from the time of the first drug administration. Red arrows indicate timing of drug administration. Errors are +/-SD.

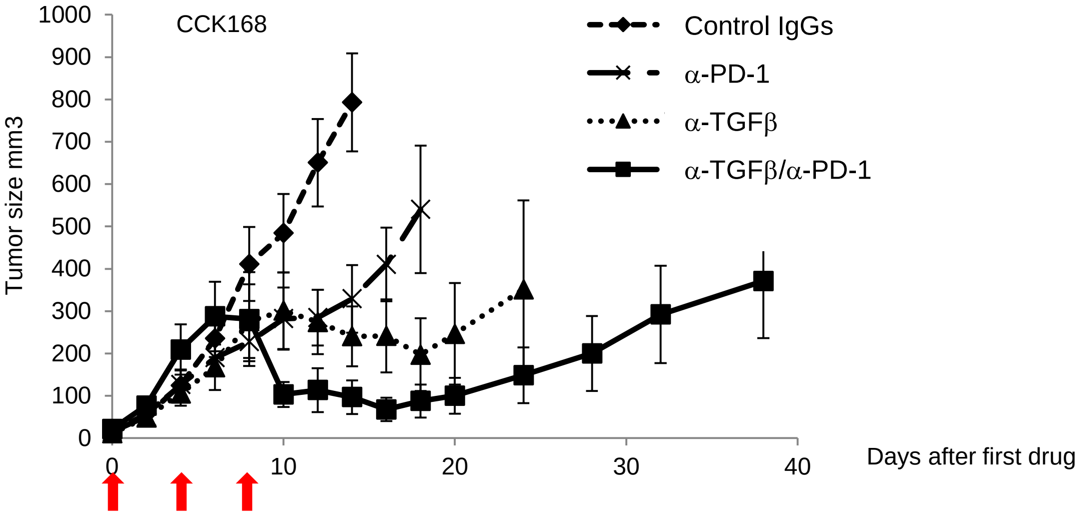


**Figure S2. CCK168 responses to anti-PD-1 and/or anti-TGFbeta** Biological replicate of Figure 3f. Tumor implantation and therapy was undertaken according to Fig.1b. Growth of CCK168 tumors (n=7-10 mice per arm) was measured every other day and average tumor volume (mm3) +/- SD was plotted. Mice were administered one of four IgG combinations, either human anti-pan-TGFbeta IgG2 (XPA.42.068) or human anti-keyhole limpet hemacyanin as control IgG2, together with rat anti-PD-1 IgG2 or rat IgG2 control. Red arrows indicate the timing of drug administration.

Growth curves represent average tumor size (mm2) per treatment group (n=7). Experiment shown is representative of three independently performed experiments. Bars represent SEM. Student t-tests: *P* (day 14, control vs α-PD1) = 0.004; *P* (day 16, α-PD1 vs α-TGFβ) = 0.05; *P* (day 16, α-PD1 vs α-PD1/α-TGFβ) = 0.007.

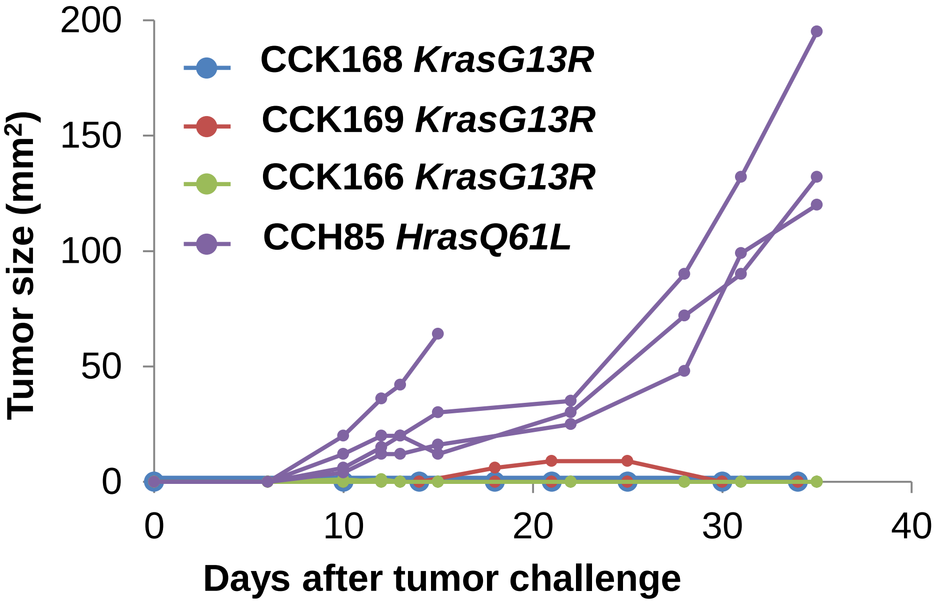


**Figure S3. anti-TGFbeta and anti-TGFbeta/anti-PD-1 combination therapy elicit long-term tumor immunity to Kras-driven tumors**. Previously CCK168-tumor bearing mice that had undergone CR in response anti-TGFbeta and or combination therapy with anti-TGFbeta/anti-PD-1 (Fig.1b,2c.d) were utilized as hosts, one month following the complete clearance of tumor, and at least 6 weeks after the last drug dose. 15, 000 tumor cells of the various lines indicated were *sc* implanted into each of five "cured" mice and five tumor-naïve mice. No additional therapy was provided. No tumor outgrowth occurred for CCK168, CCK166 and 169, despite tumor outgrowth of the same cell preparations when concurrently implanted into tumor naïve mice. In contrast, H-ras driven CCH85 tumor cells grew out normally in young "cured" and tumor naïve mice. In an additional experiment (not shown), 10/10 "cured" mice that had survived for 18 months following initial CCK168 tumor clearance, were rechallenged with CCK168 tumor, and still failed allow CCK168 tumor growth, demonstrating long term anti-tumor immunity.

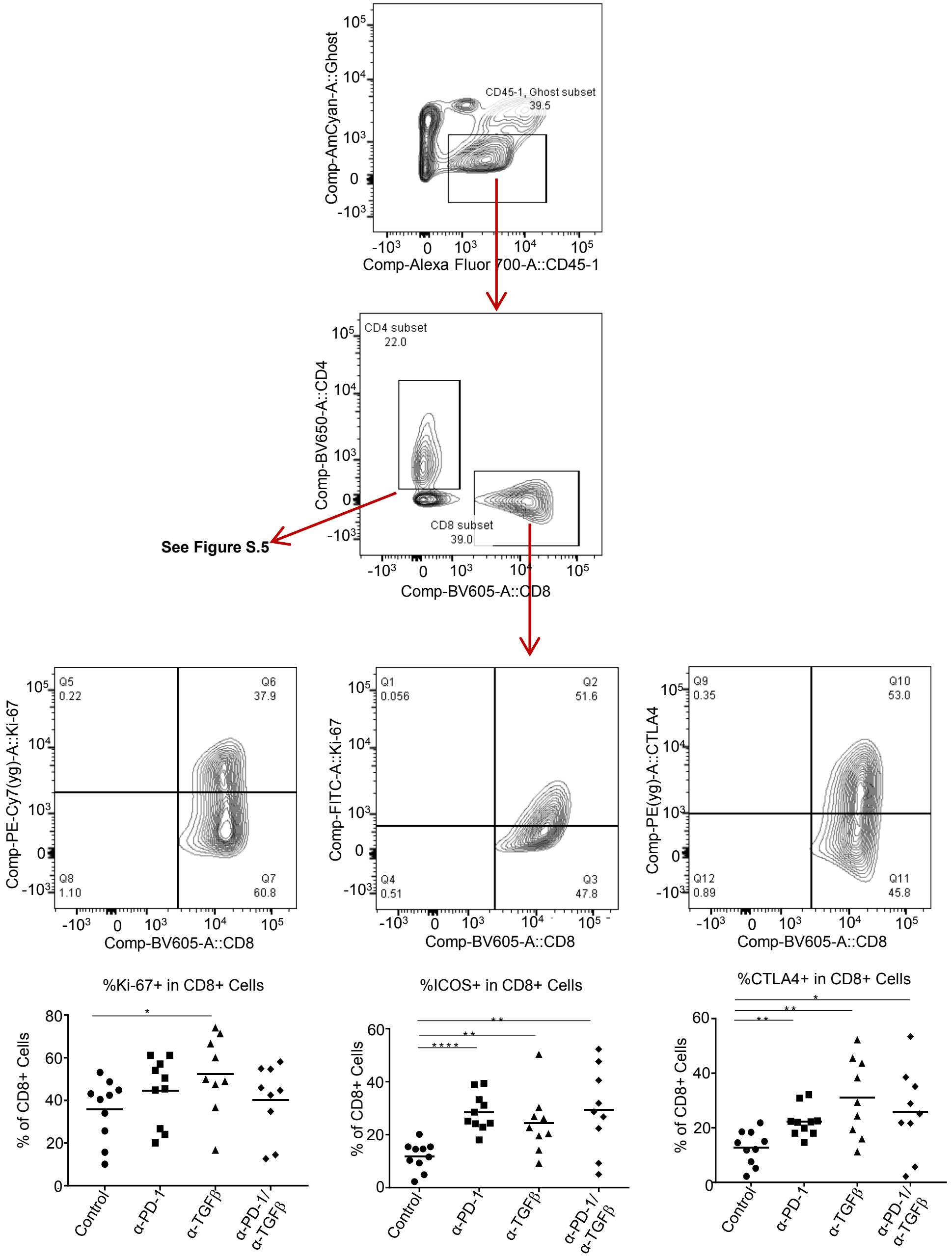


**Figure S4. Gating strategy for T cell flow cytometry and analysis of differentiation and proliferation markers.**
* =p<0.05; ** =p<0.01; *** =p<0.001

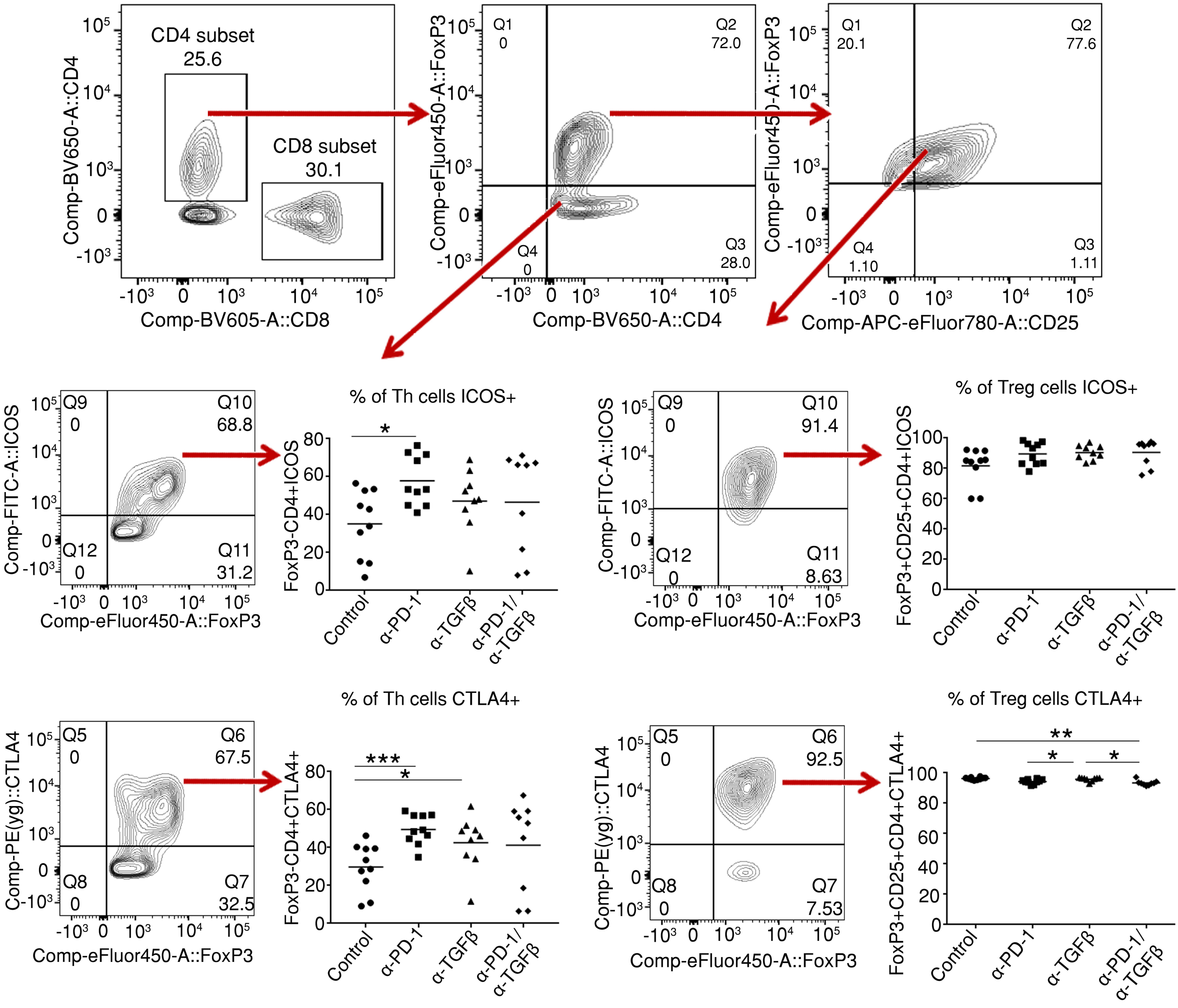


**Figure S5. Gating strategy for Th versus Treg cell flow cytometry and analysis of differentiation and proliferation markers**. * = $p<0.05$; ** = $p<0.01$; *** = $p<0.001$.

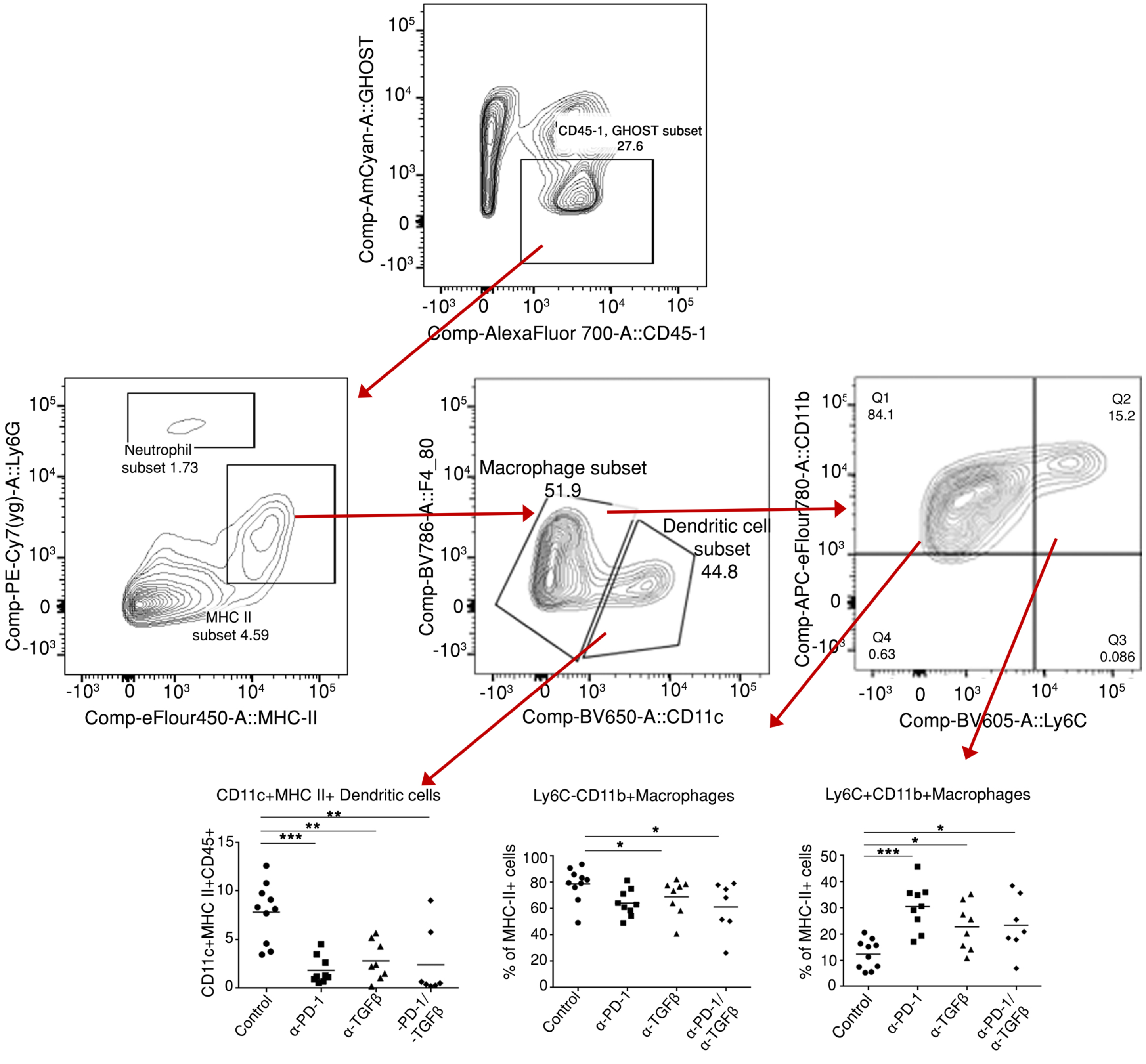


Figure S6. Gating strategy for myeloid cell flow cytometry and analysis of macrophage and dendritic cell markers. * =p<0.05; ** =p<0.01; *** =p<0.001

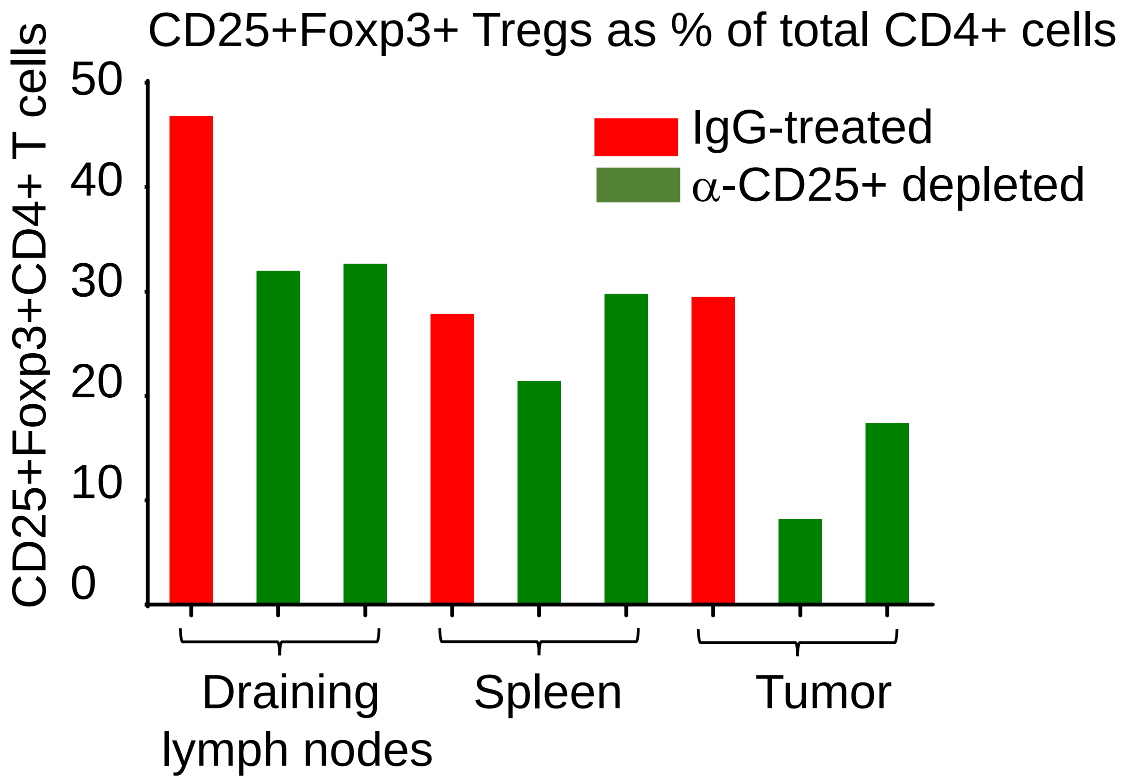


**Figure S7. Depletion of Tregs 24 hours after anti-CD25 antibody treatment.** Flow cytometry analysis was used to confirm downregulation of Tregs by anti-CD25 antibody in the animals cohorts shown in Figure 5. A few mice were euthanized 24 hours after anti-CD25 or IgG control antibody, and lymph nodes, spleen and the tumor processed and analysed for flow cytometry. Note the > 50% Treg depletion within the tumor.

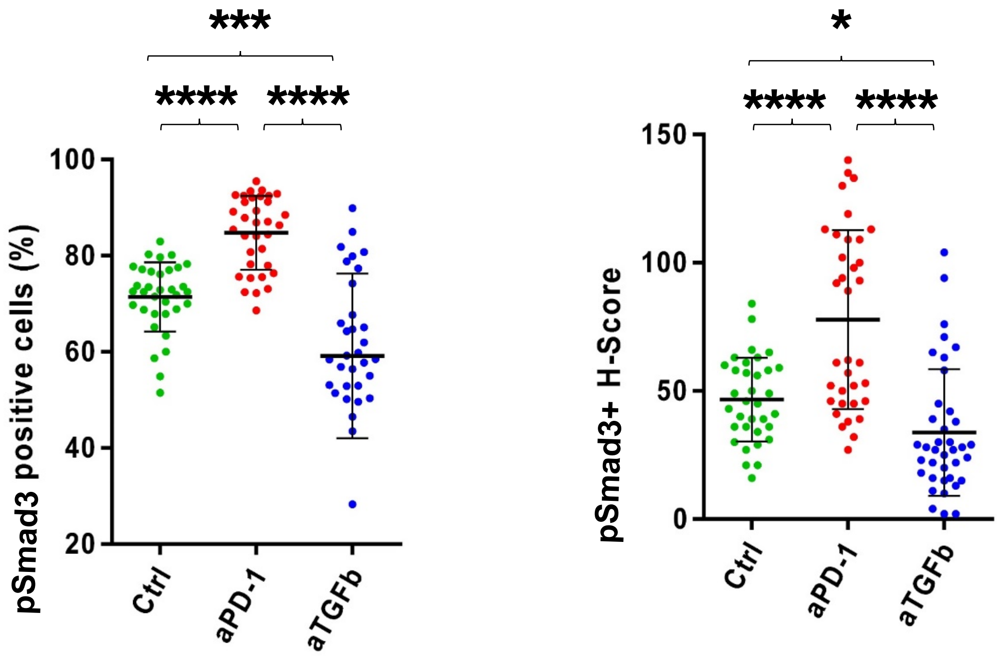


**Supplementary Figure S8. α-PD-1 induces pSmad3 in CCK168 cells.**
Independent biological replicate experiment of data shown in Figure 6g and h.

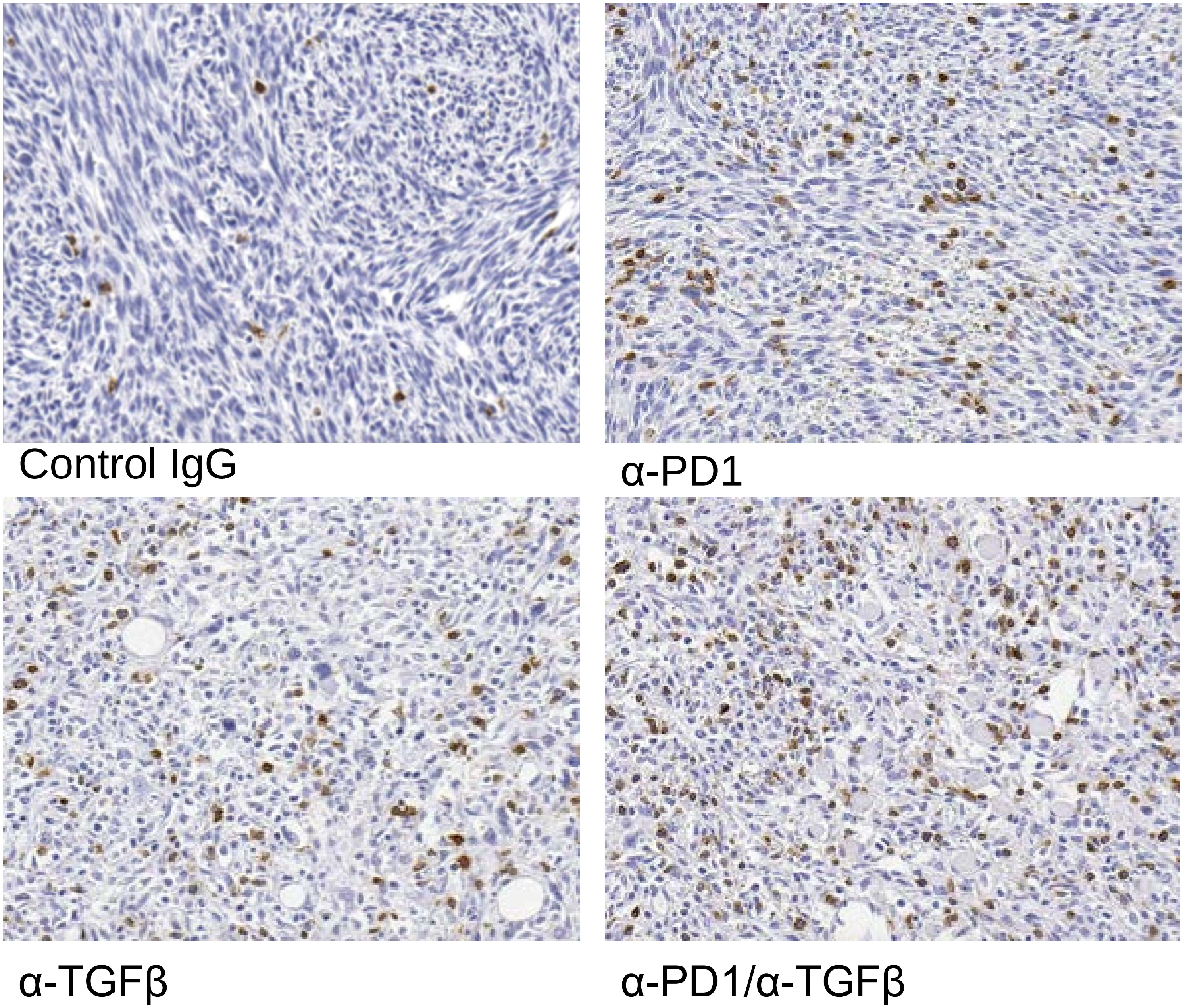


**Figure S9: CD3 T cell staining and histology of tumors after treatment with each of the four drug arms as indicate**d. Note that anti-TGFbeta-treated tumors appear less spindle in phenotype.

**Supplementary Figure S10. IHC analysis of immune infiltrates in tumors.** from each of the six SCC lines, were treated with the standard dosing regimen of IgG control antibodies or a combination $\alpha$-TGFb /$\alpha$-PD-1 therapy, and stained for total tumor leukocytes (CD45), total macrophages (CD163), T cells (CD3) or cytotoxic T cells (CD8).

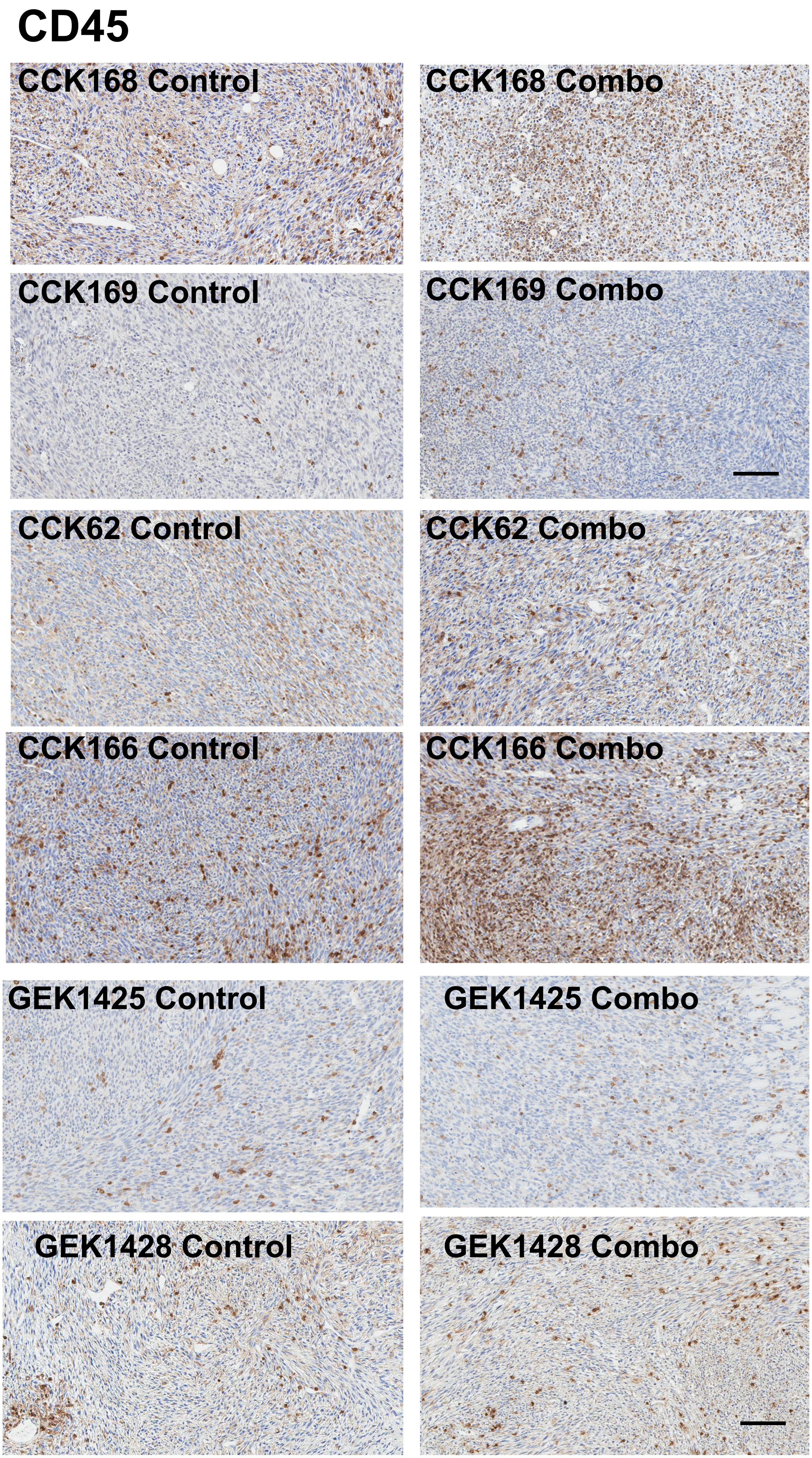
CD45
CCK168 Control
CCK168 Combo
CCK169 Control
CCK169 Combo
CCK62 Control
CCK62 Combo
CCK166 Control
CCK166 Combo
GEK1425 Control
GEK1425 Combo
GEK1428 Control
GEK1428 Combo

CD163

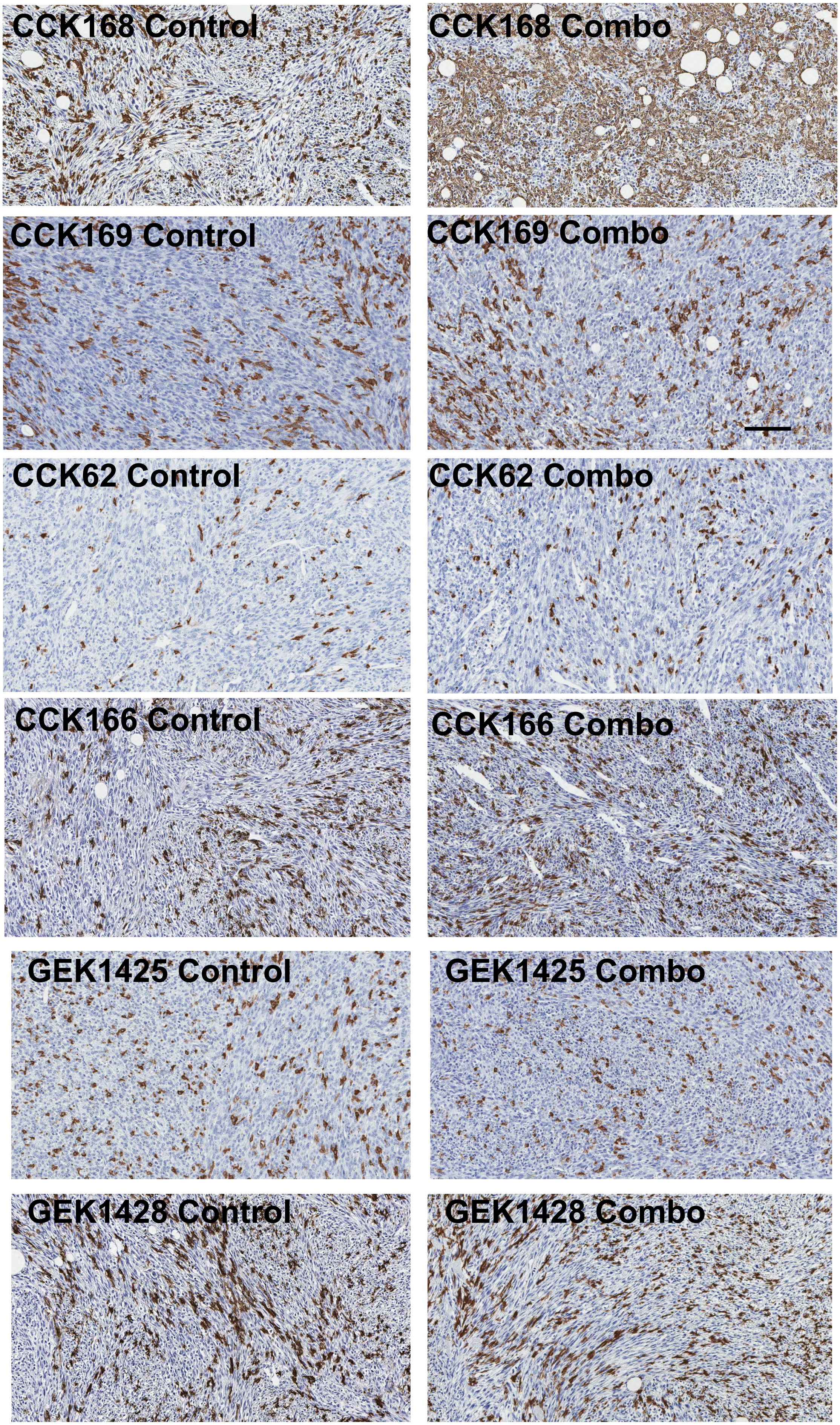

CCK168 Control
CCK168 Combo
CCK169 Control
CCK169 Combo
CCK62 Control
CCK62 Combo
CCK166 Control
CCK166 Combo
GEK1425 Control
GEK1425 Combo
GEK1428 Control
GEK1428 Combo

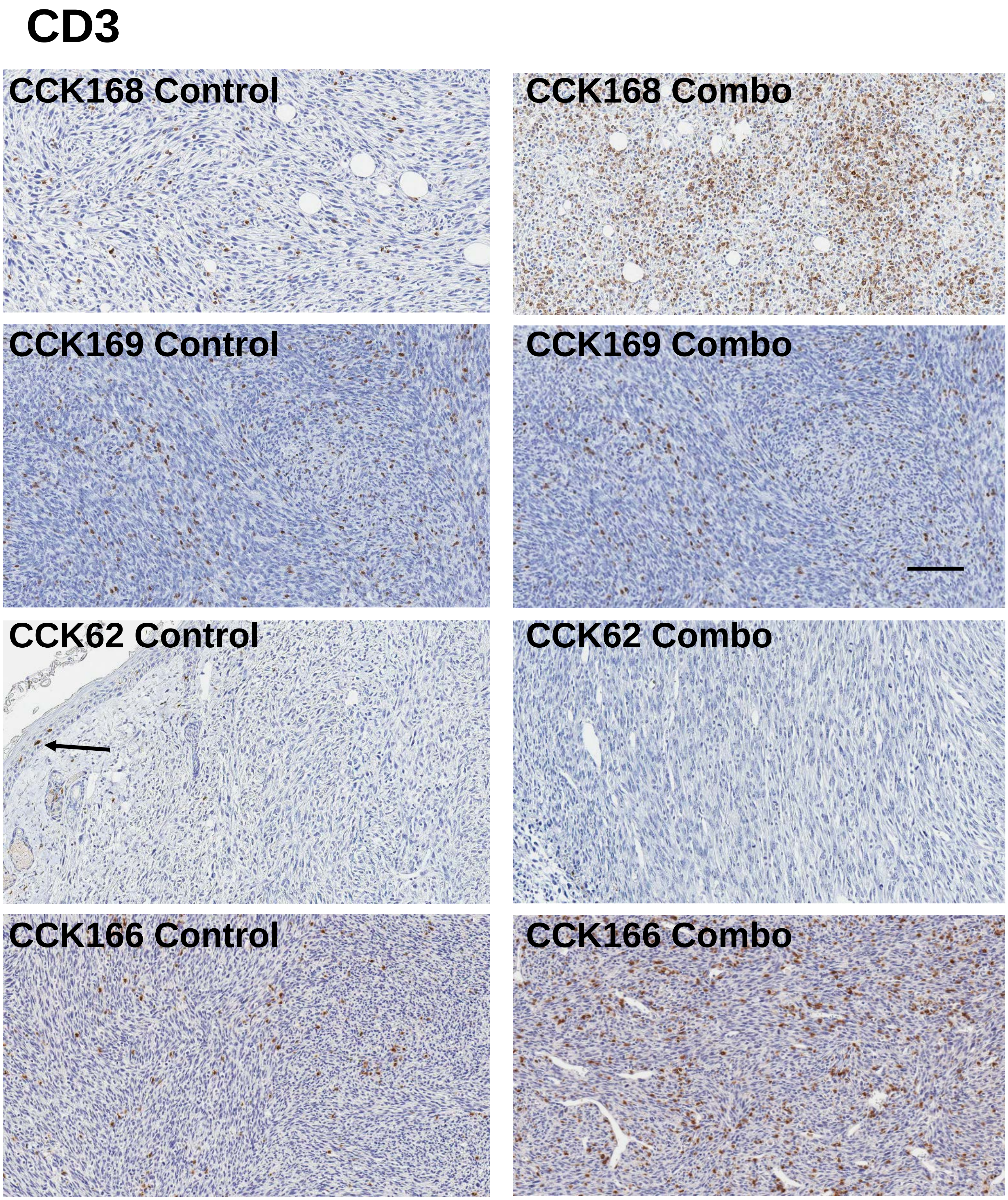
CD3
CCK168 Control
CCK168 Combo
CCK169 Control
CCK169 Combo
CCK62 Control
CCK62 Combo
CCK166 Control
CCK166 Combo

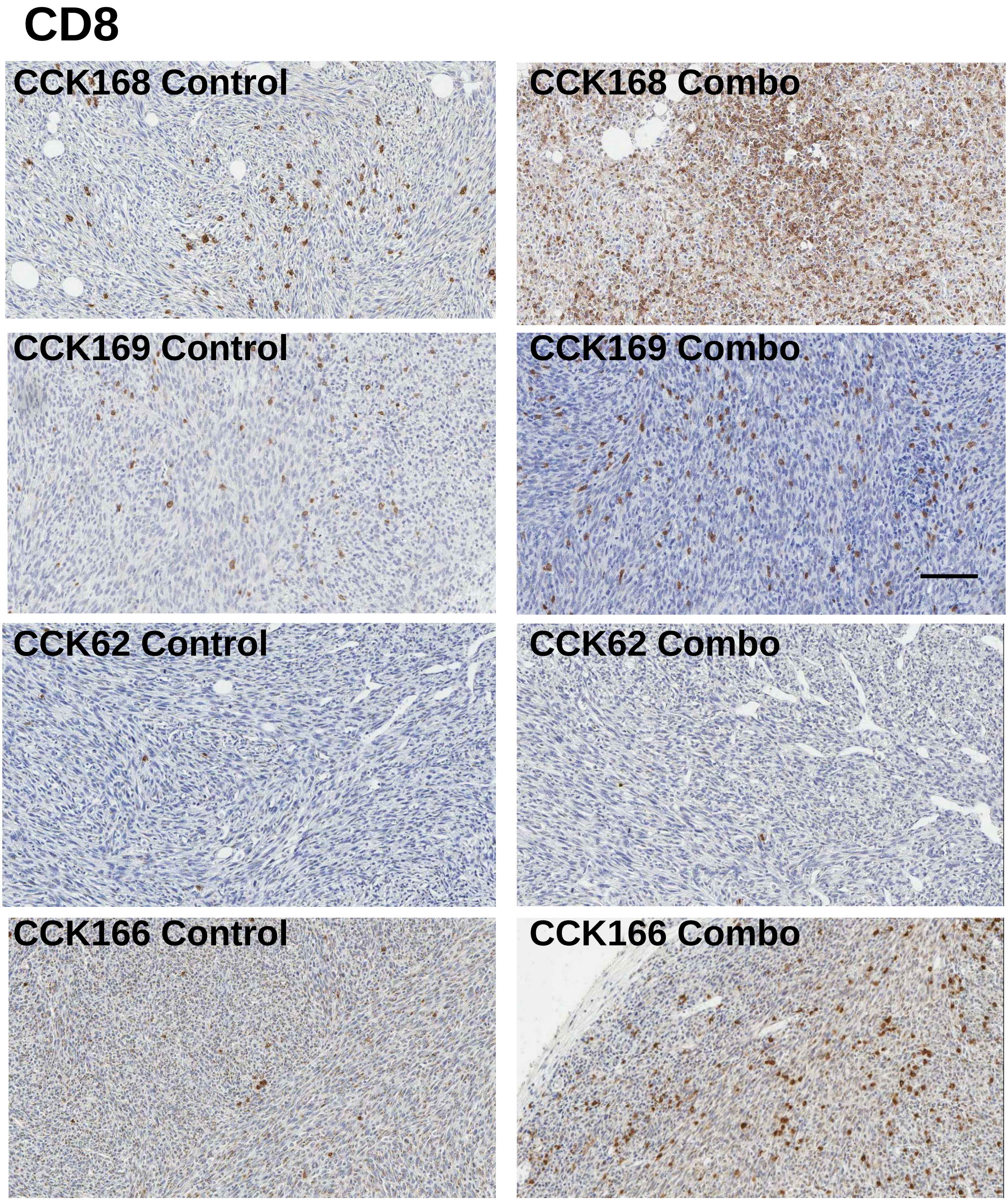
CD8
CCK168 Control
CCK168 Combo
CCK169 Control
CCK169 Combo
CCK62 Control
CCK62 Combo
CCK166 Control
CCK166 Combo
